# Immersive Virtual Reality for Pediatric Procedural Pain and Anxiety: A Structured Narrative Review and Developmentally Informed Framework for Clinical Matching and AI-Supported Personalization

**DOI:** 10.3390/nursrep16090316

**Published:** 2026-09-03

**Authors:** Domonkos Tinka, Mohammad Milad Shafaie

**Affiliations:** 1 Doctoral School of Economic and Regional Sciences, Széchenyi István University, 9026 Győr, Hungary; tinka.domonkos@sze.hu; 2 Department of Paediatrics, Petz Aladár University Teaching Hospital of Győr-Moson-Sopron County, 9024 Győr, Hungary; 3 Multidisciplinary Doctoral School of Engineering Sciences, Széchenyi István University, 9026 Győr, Hungary

**Keywords:** immersive virtual reality, pediatric procedural pain, procedural anxiety, pediatric nursing, developmental matching, clinical personalization, artificial intelligence, biofeedback, clinical implementation

## Abstract

**Background/Objectives:** Immersive virtual reality (VR) is increasingly used during pediatric procedures, but intervention effects vary across children, procedures, devices, comparators, and outcomes. This review synthesized recent evidence and developed a proposed developmentally informed child-procedure-intervention framework for clinical matching. **Methods:** A structured narrative review synthesized 96 publications identified through PubMed, Scopus, Web of Science Core Collection, the Cochrane Library, and CINAHL through 31 July 2026. Explicit searching, eligibility criteria, screening, and structured evidence characterization were used to improve transparency; however, this was not a systematic review, and no formal risk-of-bias or certainty-of-evidence assessment was performed. Evidence was interpreted according to publication type and organized by clinical context, developmental modifiers, intervention characteristics, implementation, biofeedback, and future AI-supported decision support. **Results:** The most recurrent favorable findings concerned venipuncture, intravenous access, vaccination, and related needle procedures, particularly against usual care, whereas comparisons with active distraction and evidence from oncology, burns, dentistry, orthopedics, surgery, and emergency care were more mixed. The narrative synthesis identified developmental readiness, baseline anxiety, previous procedural experience, sensory tolerance, content, interactivity, immersion, timing, comparator, concurrent analgesia, and patient preference as plausible moderators, although their predictive value remains insufficiently validated. The author-generated framework links nursing assessment, purpose-based VR selection, monitoring, stopping criteria, and clinically approved adjustment. **Conclusions:** Pediatric procedural VR should not be treated as a uniform intervention. The proposed framework is evidence-informed and hypothesis-generating, not a validated clinical decision model. Biofeedback is supported by preliminary pediatric evidence, whereas AI-supported adaptation remains a future research and translational opportunity requiring prospective validation, governance, and continuous clinical oversight.

## 1. Introduction

Pain and anxiety during clinical procedures remain an important challenge in pediatric care. Throughout childhood, patients may experience venipuncture, vaccination, intravenous (IV) cannulation, port access, burn dressing, wound care, dental anesthesia, cast removal, and emergency procedures, and many of these procedures may occur repeatedly. Although the procedure itself can be short, the emotional response is not necessarily short or minor. Fear, loss of control, previous painful experiences, parental anxiety, and anticipation may occur together at the point of care and increase the child’s distress. When this distress is not managed appropriately, its effects extend beyond immediate discomfort. Cooperation may decrease, the burden on staff and caregivers may increase, and future contact with healthcare may become more difficult or more likely to be avoided. In this context, non-pharmacological approaches that support analgesic and anxiolytic treatment without increasing medication exposure have received considerable attention. Virtual reality (VR) is one of these approaches and has been investigated as a method of distraction, engagement, and procedural support in different pediatric settings. Evidence syntheses on needle-related procedures and broader pediatric medical care generally report favorable effects on pain, fear, anxiety, or distress, although the findings are not consistent in all studies [1,2,3,4,5].

VR is clinically attractive because it can capture attention, create perceptual distance from the procedure, engage emotion, and support preparation or active coping. Pediatric applications include vaccination, venipuncture, IV placement, oncology-related vascular access, dental injections, laceration repair, and emergency care [6,7,8,9,10,11,12]. These applications are not interchangeable. Some studies report substantial reductions in pain or anxiety, whereas others show small effects or no advantage over tablet games, kaleidoscopes, bubble blowing, distraction cards, Buzzy devices, or other active comparators [13,14,15,16,17]. This heterogeneity is the central clinical problem. The relevant question is no longer simply whether VR works, but which VR format works for which child, during which procedure, and under which developmental and clinical conditions.

Previous pediatric research and evidence syntheses have commonly treated VR as a generic distraction intervention, even though developmental readiness changes how a child understands the procedure, tolerates sensory stimulation, communicates discomfort, regulates fear, interacts with clinical staff, and engages with virtual content. The same headset and software may therefore be calming for one child, overwhelming for another, and insufficiently engaging for a third. Emerging studies have examined age-related response, immersion, anxiety sensitivity, device characteristics, previous VR experience, and other patient-level modifiers [18,19,20,21,22]. Pediatric procedural VR must therefore be interpreted as a developmentally sensitive cognitive and sensory intervention, not as a uniform technology.

Artificial intelligence (AI)-enabled adaptation is not an established pediatric clinical intervention. In this review, it is treated as a future research and translational possibility motivated by documented variation in response to fixed VR. Most pediatric interventions deliver preset content for a predefined duration, with personalization limited to broad age suitability or patient choice. Cross-domain work on model-based biomechanical prediction [23] is cited only to illustrate the general technical possibility of integrating multidimensional inputs; it does not provide evidence for pediatric VR. A future clinician-facing pediatric system would therefore require its own development and validation before it could combine preprocedural characteristics with verbal, behavioral, physiological, or interaction-based inputs to recommend a small, predefined adjustment. Such inputs would not constitute diagnoses of pain or anxiety, and the system would not act autonomously. Nursing and clinical staff would retain authority over approval, continuation, modification, and termination.

Clinical value also depends on implementation. A VR intervention may reduce pain in a controlled trial yet fail in practice if setup is slow, hygiene is difficult, the device causes discomfort, staff communication is obstructed, developmentally vulnerable children are excluded, or workload is transferred to clinicians and caregivers. Studies of implementation, stakeholder experience, clinical workflow, acceptability, and resource use show that efficacy and feasibility are distinct requirements [24,25,26,27,28]. Broader evidence from technology-intensive clinical services likewise indicates that staffing, utilization, maintenance, financing, and institutional capacity influence implementation sustainability [29]. In pediatric care, nurses are central to eligibility assessment, preference elicitation, device preparation, continuous observation, stopping decisions, infection control, and workflow integration. The relevant endpoint is therefore not technological novelty but a safe, developmentally appropriate, and operationally workable intervention.

Recent systematic, scoping, umbrella, integrative, and narrative reviews describe broad patterns of reported effects, procedural concentrations, device differences, and persistent heterogeneity [1,2,3,4,5,18,30,31,32,33,34], but they do not integrate developmental readiness, therapeutic purpose, patient preference, nursing workflow, safety rules, continuous response monitoring, and bounded adaptation within one clinician-supervised pathway. This article therefore contains two deliberately separated contributions. The first is a narrative synthesis of findings reported in the reviewed literature. The second is an author-generated, evidence-informed conceptual framework that converts recurrent moderators and implementation requirements into testable clinical propositions. The framework has not undergone prospective validation and is not presented as a clinical guideline or established decision model. Four linked questions structure the review: where the clinical evidence is most consistent; which child-level and developmental factors may modify response; which intervention features permit clinically meaningful matching; and which nursing, safety, workflow, and oversight requirements should govern responsible use.

The primary review question was as follows: How can contemporary evidence on pediatric procedural VR be integrated to determine when, for whom, and under what clinical and implementation conditions VR can be appropriately matched to an individual child and procedure? Evidence on clinical effectiveness establishes where VR may provide benefit; developmental and patient-level evidence addresses for whom and under which conditions benefit may vary; intervention-format evidence addresses which delivery characteristics may support clinical fit; and safety, acceptability, implementation, and resource evidence determines whether a clinically promising intervention can be delivered responsibly in practice. Biofeedback, personalization, and AI-related evidence are included only to examine how this matching model might be extended in future research, not as evidence of current clinical effectiveness.

### Operational Terminology and Outcome Constructs

In this review, immersive VR refers to a computer-generated environment delivered through a head-mounted display that substantially replaces the user’s view of the clinical environment and supports head-tracked viewing or interaction. Semi-immersive VR refers to projected, dome-screen, or large-screen environments that create environmental engagement without complete visual occlusion. Responsive VR changes in direct response to a predefined user input. Biofeedback-based VR is a responsive subtype in which a measured physiological or behavioral signal produces visible or audible feedback. Adaptive VR changes content, intensity, pace, or difficulty according to predefined rules or model outputs. AI-supported VR is reserved here for a future system in which a trained machine-learning model integrates multiple inputs to classify or predict a clinically relevant state and generates a bounded recommendation. Deterministic threshold rules and single-signal biofeedback are not described as AI.

Immersive and semi-immersive modalities were retained because the review examines clinical matching across delivery formats rather than assuming that all VR technologies are interchangeable. Semi-immersive evidence in the primary-study corpus was limited to two non-head-mounted dome-screen interventions [35,36]. These publications are identified explicitly in Table 1 and were interpreted as a distinct delivery format; their findings were not pooled with head-mounted immersive VR. Inclusion therefore permits comparison of clinical fit while preserving the technological and evidentiary distinction between modalities. Given that semi-immersive evidence comprised only two primary publications, immersive VR remains the principal evidentiary focus of this review; the semi-immersive findings are retained only to inform delivery-format matching and are interpreted separately.

Clinical outcomes were also kept conceptually separate. Pain refers to the child’s reported or observed pain experience; fear is a response to an identifiable immediate threat; anxiety is anticipatory or diffuse apprehension; and distress is a broader emotional or behavioral response that may include fear, anxiety, agitation, or withdrawal. Cooperation describes procedural behavior and completion, not emotional comfort. Physiological responses indicate arousal or autonomic change and cannot independently identify pain, fear, or anxiety. Acceptability, feasibility, procedural success, caregiver experience, and staff workload were treated as additional and distinct implementation outcomes. A change in one construct was not interpreted as evidence of change in another unless the publication measured both.

## 2. Methodology: Structured Narrative Review and Critical Synthesis

### 2.1. Review Design and Methodological Positioning

A structured narrative review was selected because the objective was critical conceptual integration across heterogeneous primary trials, feasibility and implementation studies, qualitative research, and secondary evidence rather than estimation of one intervention effect or systematic mapping of one evidence field. A systematic review can accommodate heterogeneous designs, but it requires a prespecified protocol, reproducible search strategy, and design-appropriate critical appraisal that were not part of this project. A scoping review was also a plausible option, but the present work was not conducted under a scoping-review protocol or reporting framework and was designed to interpret relationships among clinical effects, developmental modifiers, intervention mechanisms, nursing workflow, safety, biofeedback, and a future model of personalization rather than to produce a comprehensive evidence map. We therefore retained the narrative label rather than retrospectively relabeling the review as systematic or scoping.

The terms structured and narrative are both intentional. The review used explicit databases, dates, eligibility criteria, screening stages, and a standardized evidence table to reduce the opacity of unsystematic narrative selection. These procedures improve transparency but do not convert the work into a systematic review. The review was not conducted under a registered systematic-review protocol, did not include a comprehensive grey-literature search, and did not perform formal study-level risk-of-bias assessment, certainty grading, or meta-analysis. Consequently, the methods support an auditable identification pathway and a critical narrative synthesis, but not pooled efficacy estimates, formal certainty claims, or clinical recommendations graded by evidence strength.

The review was not prospectively registered in OSF, PROSPERO, or another registry because it was designed as a structured narrative review rather than a systematic review. No claim of protocol registration is therefore made. The absence of a prospectively archived protocol limits readers’ ability to distinguish fully between decisions specified before searching and refinements made during narrative synthesis; this limitation is acknowledged explicitly.

### 2.2. Search Strategy and Eligibility Framework

This structured narrative review used an explicit search, eligibility, and synthesis framework to identify clinically and conceptually relevant evidence on immersive virtual reality (VR) for pediatric procedural pain and anxiety. Searches were conducted in PubMed, Scopus, Web of Science Core Collection, the Cochrane Library, and the Cumulative Index to Nursing and Allied Health Literature (CINAHL). English-language publications dated from January 2020 and available through 31 July 2026 were considered. The 2020 starting point was used to focus the review on the contemporary phase of pediatric clinical VR, characterized by more portable and accessible head-mounted hardware, expanding interactive content, and increasing attention to implementation in routine care; it is not presented as a universal technological breakpoint. Eligible reviews published during this period could incorporate earlier primary studies, but pre-2020 primary reports were not independently extracted. The search process was finalized on 3 August 2026. Forward and backward citation checking of recent reviews and eligible publications was also performed to confirm coverage of relevant evidence. Google Scholar was used only for citation checking and targeted retrieval and was not treated as an independent evidence source.

Across databases, the search combined terms related to virtual reality and immersive virtual reality with terms describing pediatric populations, procedural pain, anxiety, fear, distress, venipuncture, intravenous cannulation, vaccination, injections, needle-related procedures, surgery, burn and wound care, and other relevant clinical procedures. Additional combinations addressed age and developmental response, immersion, interactivity, biofeedback, implementation, nursing practice, personalization, adaptive systems, and artificial intelligence. Controlled vocabulary was used where available, and free-text terms, spelling variants, field restrictions, and search syntax were adapted to the indexing structure of each database. The same clinical eligibility framework was applied throughout the identification and screening process.

Core clinical eligibility required a pediatric population and patient-facing immersive or semi-immersive VR delivered through a head-mounted display or an equivalent immersive environment in relation to a medical procedure. Eligible outcomes or domains included pain, anxiety, fear, distress, preparation, cooperation, acceptability, safety, feasibility, and implementation. Procedures included venipuncture, intravenous cannulation, vaccination, injection, port access, oncology-related vascular access, burn and wound care, dental and otolaryngology procedures, imaging preparation, and emergency care. Eligible evidence comprised reviews, randomized and quasi-experimental studies, feasibility and pilot studies, implementation research, stakeholder studies, and safety or usability reports. Publications were excluded when they involved adults only, non-clinical VR, staff training or simulation without pediatric patient outcomes, rehabilitation unrelated to procedural pain or anxiety, vaccine hesitancy without procedural outcomes, surgical navigation, or technical systems without a direct pediatric clinical application. References [23,29,37,38] are cross-domain contextual sources used only to delimit possible technical, cognitive, resource, or institutional considerations. They were excluded from the 96-publication clinical corpus and were not used as evidence of pediatric VR effectiveness, feasibility, or safety. At the point of use, each cross-domain source is restricted to the proposition it supports: Reference [23] to the technical plausibility of multidimensional model-based prediction, Reference [38] to general cognitive-demand context, and References [29,37] to generic resource and institutional-readiness considerations.

Both authors agreed on the eligibility framework before screening and applied it to candidate publications. M.M.S. managed the searches, record organization, initial title/abstract and full-text screening, and initial extraction. D.T. cross-checked full-text eligibility decisions and all study characteristics recorded in the evidence table. Thus, the process used a two-author collaborative verification model rather than two fully independent parallel screening and extraction streams. Differences in eligibility, classification, extraction, or interpretation were identified during cross-checking and resolved through discussion and consensus, with no unresolved discrepancies; third-reviewer adjudication was therefore not required. Consistency safeguards included a shared eligibility framework, documented search dates and screening counts, standardized extraction domains, explicit publication-type classification, cross-checking of the complete evidence table, and claim-to-citation verification during synthesis. Extracted domains included publication type, country or region, population and setting, procedure, VR format, comparator or review scope, principal findings, and the main design or reporting limitation. Evidence syntheses were used to characterize the direction and recurrence of findings, whereas primary studies informed procedure-specific effects, developmental modifiers, intervention design, and implementation. Because individual trials may also appear in included reviews, reviews and primary studies were interpreted as distinct evidence levels and were not treated as independent cumulative confirmation. These procedures reduce avoidable selection, extraction, and interpretive inconsistency but do not substitute for independent duplicate review, formal risk-of-bias assessment, or certainty grading.

### 2.3. Study Selection and Evidence Organization

**Figure 1:**
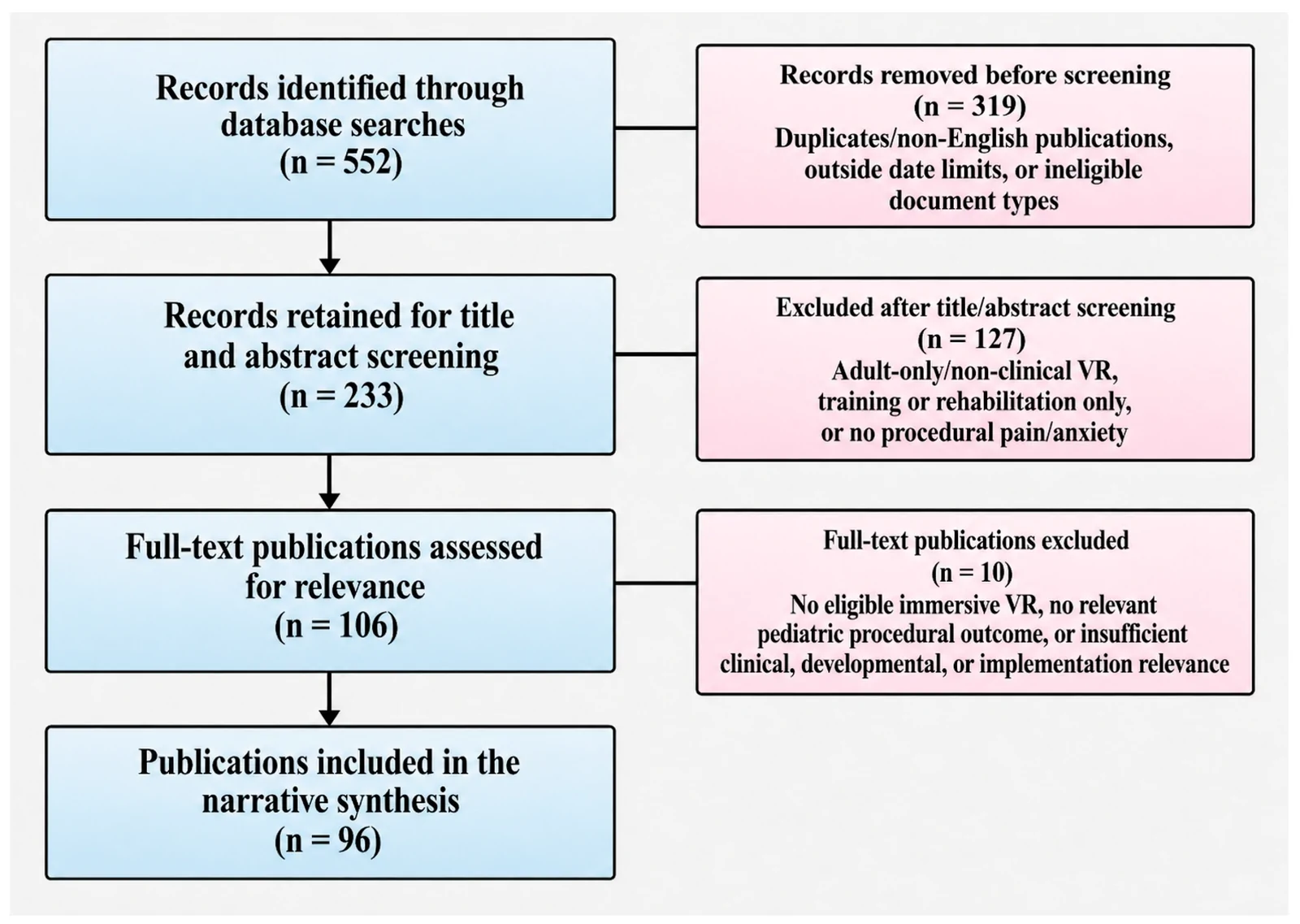
Clinical evidence-identification and selection process for the structured narrative synthesis. A total of 552 records were identified across the five bibliographic databases: 233 were retained for title and abstract screening, 106 full-text publications were assessed, and 96 publications were included in the final narrative synthesis.

The database searches identified 552 records. Before title and abstract screening, 319 records were removed as duplicates or because they fell outside the prespecified language, publication-year, or document-type criteria. The remaining 233 records were screened against the pediatric procedural and immersive-VR eligibility framework. At this stage, 127 records were excluded because they involved adult or non-clinical populations, addressed training or rehabilitation without relevant procedural outcomes, did not evaluate patient-facing immersive VR, or fell outside pediatric procedural pain and anxiety. One hundred and six full texts were assessed. Ten were excluded because they lacked an eligible immersive VR intervention, did not report a relevant pediatric procedural outcome, or did not provide clinical, developmental, or implementation information relevant to the review questions. Ninety-six publications were included in the final narrative synthesis. Figure 1 summarizes the complete evidence-selection process.

The evidence was synthesized narratively rather than statistically because the publications differed substantially in design, population, procedure, VR hardware, content, comparator, setting, outcome assessment, and developmental focus. Publications were first characterized by design and the evidentiary question each design could answer, then grouped by clinical context. Evidence syntheses informed recurrence and heterogeneity; randomized and other comparative studies informed short-term clinical effects; feasibility and pilot studies informed deliverability and short-term tolerance; implementation and qualitative studies informed workflow, acceptability, and experience; and secondary or re-analytic publications informed possible modifiers. These evidentiary roles were not treated as equivalent. Separate attention was given to evidence syntheses; needle-related procedures, vaccination, venipuncture, IV access, and injections; oncology-related vascular access; burn and wound care; other pediatric and perioperative procedures; and feasibility, implementation, acceptability, developmental, biofeedback, or adaptive evidence. Table 1 presents all 96 included publications and their main evidentiary roles. Table 2 provides a cross-publication summary of recurrent clinical domains and moderators, the direction and narrative consistency of findings, the dominant evidence basis, representative supporting publications, and the principal interpretive constraint. The labels in Table 2 are descriptive narrative judgments and must not be interpreted as formal risk-of-bias or certainty grades.

The corpus comprised 17 evidence syntheses and 79 primary or other clinically focused publications. Table 1 organizes these publications by dominant analytic role: 38 concerned needle-related, vaccination, injection, venipuncture, or intravenous procedures; six concerned oncology-related vascular access; three concerned burn care; eight concerned other procedural settings; 15 focused principally on feasibility, implementation, acceptability, developmental modifiers, biofeedback, or adaptive features; and nine were recent primary publications addressing emerging clinical questions. These mutually exclusive table groups support traceability, while the narrative themes in Section 3, Section 4, Section 5, Section 6 and Section 7 were generated by comparing recurrent findings across groups. Clinical-effect statements were anchored primarily in evidence syntheses and comparative studies; feasibility, acceptability, safety, usability, workflow, and contextual findings were used only for those respective questions.

Among the 79 non-review publications, 78 single-country publications spanned 27 countries and one trial was multinational (Poland, Spain, Ireland, and the United Kingdom). The largest concentrations were in Türkiye (18 publications), the United States (10), and Canada (8), followed by Spain and South Korea (4 each); all other countries contributed three or fewer publications. This distribution indicates broad international interest but uneven representation. Transferability may be limited by differences in pediatric procedural practice, staffing and nursing roles, technology access, language and content, infection-control requirements, financing, and health-system workflow. The framework should therefore be prospectively validated and locally adapted rather than assumed to transfer unchanged across settings.

**Table 1:** Characteristics and key contributions of the literature included in the narrative synthesis.

No.	Author(s), Year	Study Design/ Evidence Type	Study Country/ Region	Population/ Setting	Procedure/ Clinical Context	VR Approach/ Comparator or Review Scope	Main Findings	Key Limitation/ Gap
**A. Reviews and evidence syntheses**								
1	Gök et al. (2026) [30]	Umbrella review/re-analysis	Multinational evidence synthesis	children/adolescents; multiple clinical settings	Pediatric medical procedures	Evidence synthesis of VR interventions	Across six meta-analyses, VR significantly reduced needle-related pain, although heterogeneity and review overlap were high.	Overlapping, heterogeneous reviews; little age-stratified evidence.
2	Huang et al. (2026) [5]	Scoping review	Multinational evidence synthesis	children/adolescents; multiple clinical settings	Pediatric medical procedures	Evidence synthesis of VR interventions	Most of 22 studies favored VR for procedural pain/distress versus standard care; evidence was short-term and heterogeneous.	No pooled effects or certainty grading; mostly short-term studies.
3	Shi et al. (2026) [18]	Network meta-analysis and systematic review	Multinational evidence synthesis	children/adolescents undergoing procedural care	Needle-related pediatric procedures	Comparison of immersive VR device types	Pairwise meta-analysis significantly favored VR for needle pain; device rankings varied and indirect comparisons were sparse.	Sparse indirect comparisons and inconsistent device reporting.
4	Lou et al. (2025) [39]	Systematic review and meta-analysis	Multinational evidence synthesis	pediatric burn patients	Burn/wound care procedures	Evidence synthesis of VR interventions	VR significantly reduced burn-care pain, anxiety, pain-focused attention, and heart rate; oxygen saturation did not differ.	Few small studies and heterogeneous burn-care protocols.
5	Cáceres-Matos et al. (2024) [3]	Systematic review and meta-analysis	Multinational evidence synthesis	children/adolescents undergoing procedural care	Needle-related pediatric procedures	Evidence synthesis of VR interventions	Pooled analyses significantly favored VR for needle-related pain, anxiety, and fear, with substantial heterogeneity.	Heterogeneous VR formats, comparators, outcomes, and age ranges.
6	Sánchez-Caballero et al. (2024) [4]	Systematic review	Multinational evidence synthesis	children/adolescents; multiple clinical settings	Pediatric medical procedures	Evidence synthesis of VR interventions	Across 12 RCTs, immersive VR generally reduced procedure-related pain and/or anxiety, with heterogeneous effects.	Clinical and methodological heterogeneity; sparse age-stratified evidence.
7	Tsitsi et al. (2024) [40]	Systematic review	Multinational evidence synthesis	children/adolescents with cancer	Oncology-related vascular access	Evidence synthesis of VR interventions	Five of seven oncology studies significantly reduced physical distress; psychological distress improved significantly in two.	Small heterogeneous evidence base; limited repeated-use analysis.
8	Wei et al. (2024) [41]	Meta-analysis	Multinational evidence synthesis	Children undergoing venipuncture	Venipuncture/vein puncture	Evidence synthesis of VR interventions	Meta-analysis of 10 RCTs found significant reductions in child- and caregiver-reported venipuncture pain and anxiety.	Heterogeneous VR formats, comparators, outcomes, and age ranges.
9	Gao et al. (2023) [2]	Systematic review and meta-analysis	Multinational evidence synthesis	children/adolescents undergoing procedural care	Needle-related pediatric procedures	Evidence synthesis of VR interventions	Meta-analysis of 27 RCTs found significant reductions in needle-related pain, anxiety, and fear with VR.	Heterogeneous VR formats, comparators, outcomes, and age ranges.
10	Jenabi et al. (2023) [42]	Systematic review and meta-analysis	Multinational evidence synthesis	children/adolescents undergoing procedural care	Needle-related pediatric procedures	Evidence synthesis of VR interventions	Meta-analysis of nine controlled studies found significantly lower IV-injection pain with VR; anxiety and fear were not pooled.	Heterogeneous VR formats, comparators, outcomes, and age ranges.
11	Merino-Lobato et al. (2023) [43]	Systematic review and meta-analysis	Multinational evidence synthesis	children/adolescents undergoing procedural care	Venipuncture/blood draw	VR compared with other distraction methods	VR and Buzzy significantly reduced venipuncture pain and anxiety versus standard care; neither was significantly superior.	Heterogeneous VR formats, comparators, outcomes, and age ranges.
12	Lluesma-Vidal et al. (2022) [1]	Systematic review and meta-analysis	Multinational evidence synthesis	children/adolescents undergoing procedural care	Needle-related pediatric procedures	Evidence synthesis of VR interventions	Meta-analysis found significant reductions in needle-related pain and fear with VR; methodological limitations were common.	Heterogeneous VR formats, comparators, outcomes, and age ranges.
13	Wang et al. (2022) [44]	Review	Multinational evidence synthesis	children/adolescents undergoing procedural care	Needle-related pediatric procedures	Evidence synthesis of VR interventions	Meta-analysis found significant reductions in needle-related pain, fear, and anxiety across child and observer reports.	Heterogeneous VR formats, comparators, outcomes, and age ranges.
**B. Needle, vaccination, venipuncture, IV, and injection procedures**								
14	Ali et al. (2026) [8]	Randomized controlled trial	Canada	children aged 6–17 years; ED	IV placement/cannulation	Commercial HMD VR distraction	Commercial VR was not significantly superior to standard care for IV-cannulation distress, pain, or fear.	Active comparator and short follow-up limited incremental-effect estimates.
15	Gülduran et al. (2026) [45]	Randomized controlled trial	Türkiye	children aged 5 to 12 years; ED	Intramuscular injection	VR compared with other distraction methods	Distraction methods significantly differed for injection pain, but not for fear or anxiety; VR reduced pain.	Short-term, single-setting evidence; no powered subgroup analysis.
16	Güray et al. (2026) [46]	Quasi-randomized controlled study	Türkiye	children aged 5–12 years	Venipuncture/blood draw	Immersive VR distraction	VR significantly reduced child-reported venipuncture pain and fear versus control.	Nonrandomized allocation and residual confounding.
17	Sanad et al. (2026) [7]	Quasi-experimental study	Bahrain	children aged 6–12 years; outpatient	Venipuncture/blood draw	Immersive VR distraction	Pain was significantly lower with VR than routine care; fear and distress also decreased significantly from pre- to post-venipuncture within the VR group.	Nonrandomized allocation and residual confounding.
18	Zengin et al. (2026) [13]	Randomized clinical trial	Türkiye	children aged 6–12 years	IV placement/catheterization	VR compared with other distraction methods	VR significantly reduced pain and anxiety; fear fell with VR and bubble play; procedure and crying times differed.	Short-term, single-setting evidence; no powered subgroup analysis.
19	De La Cruz Herrera et al. (2025) [6]	Randomized clinical trial	Spain	pediatric patients/children; primary care	Vaccination/immunization	Immersive VR distraction	Immersive VR significantly reduced vaccination pain and anxiety, lowered heart rate, and increased guardian satisfaction.	Short-term, single-setting evidence; no powered subgroup analysis.
20	Sánchez-López et al. (2025) [47]	Randomized controlled trial	Spain	pediatric patients/children	Vaccination/immunization	Immersive VR distraction	Immersive VR significantly reduced vaccination pain and fear and increased parental satisfaction.	Short-term, single-setting evidence; no powered subgroup analysis.
21	Vitor et al. (2025) [48]	Pilot randomized trial	Brazil	pediatric patients/children	Vaccination/immunization	Immersive VR distraction	Pilot feasibility was supported; 74% with VR versus 24% of controls reported no pain.	Pilot sample; underpowered for definitive efficacy or subgroup effects.
22	da Rocha et al. (2025) [49]	Randomized clinical trial	Brazil	children aged 4 to 14 years; ED	IV placement/catheterization	Immersive VR distraction	VR significantly reduced IV-cannulation pain and distress; distress effects were stronger in children aged 4–8 years.	Short-term, single-setting evidence; no powered subgroup analysis.
23	Czub et al. (2024) [50]	Randomized controlled trial	Multinational (Poland/Spain/Ireland/UK)	children aged 5–9 years	Venipuncture/blood draw	Interactive/active VR distraction	VR and identical non-VR distraction similarly improved needle pain/distress versus usual care; formats did not differ significantly.	Short-term, single-setting evidence; no powered subgroup analysis.
24	Sarı et al. (2024) [51]	Randomized controlled trial	Türkiye	pediatric patients/children	Vaccination/immunization	VR compared with other distraction methods	Kaleidoscope yielded significantly lower post-vaccination pain, fear, and anxiety than both VR and control.	Short-term, single-setting evidence; no powered subgroup analysis.
25	Gil et al. (2024) [52]	Randomized clinical trial	Spain	children aged 7–12 years; primary care	Venipuncture/blood draw	Immersive VR distraction	VR significantly reduced venipuncture pain and increased family satisfaction; the between-group difference in anxiety was not significant.	Short-term, single-setting evidence; no powered subgroup analysis.
26	Gold et al. (2024) [19]	Secondary analysis of RCT data	United States	children aged 10–21 years; infusion center	Venipuncture/blood draw	Immersive VR distraction	Immersion interacted with anxiety sensitivity for postprocedural anxiety; no other tested moderator significantly affected pain/anxiety.	Exploratory analysis underpowered for moderator effects.
27	Gómez-Neva et al. (2024) [53]	Clinical study	Colombia	children and adolescents; ED	Venipuncture/blood draw	Immersive VR distraction	VR did not significantly or clinically reduce venipuncture pain versus control.	Limited confounding control and no long-term follow-up.
28	Lee et al. (2024) [54]	Randomized clinical trial	South Korea	children aged 4–8 years	Venipuncture/blood draw	VR education and preparation	VR education significantly reduced observed pain/distress versus identical tablet education; procedural outcomes did not differ.	Narrow developmental range and short follow-up.
29	Makhijani et al. (2024) [55]	Pilot feasibility study	Australia	children aged 4–14 years	Vaccination/immunization	Immersive VR procedural support during immunization	VR was feasible and acceptable in needle-phobic children; 90% were immunized, but efficacy was not established.	Small feasibility sample; clinical efficacy not established.
30	Zavlanou et al. (2024) [15]	Randomized clinical trial	Switzerland	pediatric patients/children	Venipuncture/blood draw	Interactive VR game versus the same game delivered on a tablet	Pain and anxiety were low with both VR and tablet; no primary or secondary outcome differed significantly.	Active comparator and short follow-up limited incremental-effect estimates.
31	Ferraz-Torres et al. (2023) [56]	Quasi-experimental study	Spain	pediatric patients/children; ED	Venipuncture/blood draw	Immersive VR distraction	VR was associated with significantly lower venipuncture pain and anxiety and reduced parental blocking responses.	Nonrandomized allocation and residual confounding.
32	Lee et al. (2023) [35]	Randomized clinical trial	South Korea	young children; ED	IV placement/catheterization	Non-HMD domed-ceiling VR distraction	Domed-ceiling VR significantly reduced overall FLACC pain/distress during IV placement versus standard care.	Single-device, single-setting evidence; limited generalizability.
33	Orhan et al. (2023) [57]	Randomized controlled trial	Türkiye	children aged 7–12 years; outpatient	Venipuncture/blood draw	Immersive VR distraction	VR significantly reduced post-venipuncture pain and intraprocedural heart rate; oxygen-saturation change was not significant.	Short-term, single-setting evidence; no powered subgroup analysis.
34	Wong et al. (2023) [58]	Randomized clinical trial	Hong Kong	children aged 4 to 12 years	Venipuncture/blood draw	Immersive VR distraction	Immersive VR significantly reduced pain and anxiety, increased clinician satisfaction, and shortened venipuncture duration.	Short-term, single-setting evidence; no powered subgroup analysis.
35	van den Berg et al. (2023) [59]	Non-inferiority randomized trial	Netherlands	children aged 6 to 18 years	Pediatric treatment stress/needle procedures	VR hypnosis/hypnoanalgesia	VR hypnosis was non-inferior to medical hypnosis for patient-reported pain and fear; secondary outcomes were comparable.	Active comparator and short follow-up limited incremental-effect estimates.
36	Atzori et al. (2022) [60]	Clinical study	Italy	children aged 7–11 years	Venipuncture/blood draw	Immersive VR distraction	VR significantly reduced venipuncture pain intensity and unpleasantness; time thinking about pain did not differ.	Limited confounding control and no long-term follow-up.
37	Hsu et al. (2022) [61]	Randomized controlled trial	Taiwan	children aged 6–12 years	IV placement/catheterization	Interactive VR play versus routine care	Interactive VR significantly reduced child- and caregiver-rated IV-placement pain and fear; insertion time did not differ.	Short-term, single-setting evidence; no powered subgroup analysis.
38	Ryu et al. (2022) [62]	Randomized clinical trial	South Korea	pediatric patients/children	Venipuncture/blood draw	VR education and preparation	VR education significantly reduced observed pain/anxiety, increased parental satisfaction, and reduced procedural difficulty.	Short-term, single-setting evidence; no powered subgroup analysis.
39	Thybo et al. (2022) [63]	Randomized controlled trial	Denmark	children aged 4–7 years	Venous cannulation	Interactive VR versus smartphone/tablet distraction	VR was acceptable but did not significantly reduce cannulation pain versus smartphone/tablet distraction.	Short-term, single-setting evidence; no powered subgroup analysis.
40	Canares et al. (2021) [64]	Pilot randomized controlled trial	United States	Pediatric patients and young adults aged 7–22 years; ED	Venipuncture/blood draw	Immersive VR distraction	VR improved observed coping, but patient distress and pain did not differ significantly between groups.	Pilot sample; underpowered for definitive efficacy or subgroup effects.
41	Erdogan et al. (2021) [14]	Randomized controlled trial	Türkiye	children aged 7–12 years	Venipuncture/blood draw	VR compared with other distraction methods	VR, distraction cards, and Buzzy significantly reduced venipuncture pain and anxiety versus control; Buzzy scored lowest.	Short-term, single-setting evidence; no powered subgroup analysis.
42	Goldman et al. (2021) [65]	Randomized controlled trial	Canada	children aged 6–16 years; ED	IV placement/catheterization	Immersive VR distraction	VR did not significantly improve pain or postprocedure anxiety, but increased satisfaction and enjoyment.	Short-term, single-setting evidence; no powered subgroup analysis.
43	Kumari et al. (2021) [12]	Clinical study	India	pediatric patients/children; dental clinic	Dental/intraoral injection	Interactive/active VR distraction	Immersive VR significantly reduced dental-injection pain and postprocedure anxiety versus non-immersive VR.	Limited confounding control and no long-term follow-up.
44	Lee et al. (2021) [36]	Pilot randomized controlled trial	South Korea	children aged 2 to 6 years; ED	IV placement	Non-HMD dome-screen VR distraction	Dome-screen VR was feasible; pain was lower than control but not significantly different.	Pilot sample; underpowered for definitive efficacy or subgroup effects.
45	Litwin et al. (2021) [66]	Pilot randomized controlled trial	Canada	children aged 8 to 17 years; ED	IV placement/catheterization	Immersive VR versus tablet/video distraction	VR produced clinically meaningful pain reduction; fear/distress were not significantly different, and heart-rate increase was greater.	Pilot sample; underpowered for definitive efficacy or subgroup effects.
46	Osmanlliu et al. (2021) [67]	Pilot randomized controlled trial	Canada	pediatric patients/children; ED	IV placement/catheterization	Interactive/active VR distraction	VR was feasible; self-reported pain was not significantly different, while exploratory proxy distress/pain-memory outcomes favored VR.	Pilot sample; underpowered for definitive efficacy or subgroup effects.
47	Chen et al. (2020) [68]	Randomized trial	Taiwan	children aged 7–12 years; ED	IV placement/catheterization	Immersive VR distraction	VR significantly reduced child-, caregiver-, and nurse-rated IV-insertion pain and fear and shortened successful insertion time.	Short-term, single-setting evidence; no powered subgroup analysis.
48	Koç et al. (2020) [69]	Randomized controlled trial	Türkiye	pediatric patients/children	Venipuncture/blood draw	VR compared with other distraction methods	Both VR and kaleidoscope significantly reduced venipuncture pain and anxiety versus control.	Short-term, single-setting evidence; no powered subgroup analysis.
49	Piskorz et al. (2020) [70]	Three-group experimental study	Poland	pediatric patients/children	Venipuncture/blood draw	Active VR versus passive VR versus control	Active and passive VR significantly reduced pain; stress fell significantly only with active VR; VR formats did not differ.	Limited confounding control and no long-term follow-up.
50	Özalp et al. (2020) [71]	Randomized controlled trial	Türkiye	children aged 5–12 years	Venipuncture/blood draw	Immersive VR distraction	Both VR environments reduced blood-draw pain; postprocedure fear and anxiety differed significantly versus control.	Short-term, single-setting evidence; no powered subgroup analysis.
51	Wong et al. (2020) [72]	Controlled clinical study	United States	pediatric patients/children	PIV insertion and PICC placement or dressing change	Interactive/active VR distraction	Postprocedure pain was lower with VR than routine care, but the between-group difference was not significant.	Limited confounding control and no long-term follow-up.
**C. Oncology-related vascular access**								
52	Silva et al. (2025) [73]	Clinical study	Brazil	children and adolescents; oncology/hematology	Oncology-related needle/vascular access	Interactive/active VR distraction	Within-patient VR significantly reduced heart rate, pain, agitation, and crying during venous/catheter procedures.	Limited confounding control and no long-term follow-up.
53	Reitze et al. (2024) [74]	Randomized controlled trial	Germany	children aged 6–18 years; oncology/hematology, outpatient	Pediatric oncology procedures	Immersive VR distraction	VR significantly reduced peri-interventional pain, anxiety, and distress versus standard care.	Short-term, single-setting evidence; no powered subgroup analysis.
54	Amali et al. (2023) [75]	Quasi-experimental study	India	pediatric patients/children; oncology/hematology	Oncology-related needle/vascular access	Immersive VR distraction	VR significantly reduced child- and mother-rated IV-cannulation pain and fear versus control.	Nonrandomized allocation and residual confounding.
55	Hundert et al. (2022) [76]	Pilot randomized controlled trial	Canada	children aged 8 to 18 years; oncology/hematology	Port/implanted vascular access	Interactive/active VR distraction	VR was feasible; pain/distress favored VR without statistical significance, and fear was similar to iPad distraction.	Pilot sample; underpowered for definitive efficacy or subgroup effects.
56	Gerçeker et al. (2021) [9]	Randomized controlled trial	Türkiye	children and adolescents; oncology/hematology	Port/implanted vascular access	Immersive VR distraction	VR significantly reduced port-access pain, fear, and anxiety versus control.	Short-term, single-setting evidence; no powered subgroup analysis.
57	Semerci et al. (2021) [10]	Randomized controlled trial	Türkiye	children aged 7 to 18 years; oncology/hematology	Port/implanted vascular access	Commercial HMD VR distraction	VR significantly reduced child- and parent-rated pain during venous port access versus control.	Short-term, single-setting evidence; no powered subgroup analysis.
**D. Burn and wound care**								
58	Jeffs et al. (2024) [77]	Randomized controlled trial	United States	children and adolescents; burn care	Burn/wound care procedures	Interactive/active VR distraction	VR was not significantly superior to nursing standard care for procedural pain; patient/procedure factors influenced pain.	Short-term, single-setting evidence; no powered subgroup analysis.
59	Wang et al. (2023) [78]	Clinical study	China	school-aged children; burn care	Burn/wound care procedures	Interactive/active VR distraction	Within-child VR reduced Wong-Baker and FLACC pain during and after burn dressing versus non-VR dressing.	Limited confounding control and no long-term follow-up.
60	Khadra et al. (2020) [79]	Within-subject randomized crossover trial	Canada	young children; burn care	Burn/wound care procedures	Non-HMD or hybrid VR environment	Hybrid VR significantly reduced FLACC pain and increased comfort; nurse-rated numeric pain was not significantly different.	Small short-term design; limited generalizability.
**E. Other pediatric procedures and clinical settings**								
61	Ciolini et al. (2026) [80]	Randomized controlled trial	Italy	children aged 3–17 years; outpatient	Pediatric procedural care	Immersive VR distraction	VR significantly reduced procedural pain but not preprocedural anxiety; maternal anxiety predicted child anxiety.	Narrow developmental range and short follow-up.
62	Guingo et al. (2026) [17]	Randomized clinical trial	Canada	children aged 6–17 years; outpatient, orthopedic clinic	Orthopedic outpatient procedures	Immersive VR versus tablet/video distraction	VR was not significantly superior to tablet games overall; adolescents aged ≥13 years had significantly lower anxiety.	Short-term, single-setting evidence; no powered subgroup analysis.
63	Elhalafawy et al. (2025) [81]	Randomized controlled trial	Egypt	children aged 6–12 years	Pediatric procedural care	VR compared with other distraction methods	VR, Buzzy, and kaleidoscope significantly reduced pain, fear, and anxiety versus control; VR produced the largest pain reduction.	Short-term, single-setting evidence; no powered subgroup analysis.
64	Felnhofer et al. (2025) [82]	Randomized controlled trial	Austria	children aged 10–17 years	Ingrown toenail surgery	Immersive VR distraction	No overall self-reported pain/stress benefit occurred; both VR conditions reduced heart rate during anesthesia.	Active comparator and short follow-up limited incremental-effect estimates.
65	McEvoy et al. (2025) [11]	Clinical study	United States	children aged 6–17 years; ED	Laceration repair	Interactive/active VR distraction	VR significantly reduced pain and fear and physical holding; procedure duration did not differ significantly.	Limited confounding control and no long-term follow-up.
66	Fabricant et al. (2024) [16]	Randomized controlled trial	United States	pediatric patients/children; outpatient, orthopedic clinic	Cast removal	Immersive VR versus tablet/video distraction	Interactive VR was not superior to VR video or tablet video for heart rate, pain, or anxiety.	Active comparator and short follow-up limited incremental-effect estimates.
67	Gilboa et al. (2024) [83]	Prospective randomized trial	Israel	Pediatric surgical patients	Common pediatric surgical procedures	Infection-control-compatible VR goggles	Fear/anxiety decreased significantly within VR, but postprocedure between-group differences were not significant amid baseline imbalance.	Short-term, single-setting evidence; no powered subgroup analysis.
68	Jivraj et al. (2020) [84]	Randomized controlled trial	Canada	pediatric patients/children	Cast removal	Immersive VR distraction	VR significantly reduced intraprocedural and postprocedural anxiety during cast removal and was well tolerated.	Short-term, single-setting evidence; no powered subgroup analysis.
**F. Feasibility, implementation, acceptability, and age/adaptive evidence**								
69	Karppa et al. (2026) [85]	Feasibility study	Finland	pediatric patients/children	Venipuncture/needle procedure	VR-guided breathing/natural environment	VR-guided breathing significantly lowered respiratory rate and increased parasympathetic HRV activity; no adverse effects occurred.	Small feasibility sample; clinical efficacy not established.
70	Stålberg et al. (2026) [24]	Qualitative/implementation study	Sweden	pediatric patients/children	Pediatric treatment stress/needle procedures	Immersive VR distraction	Nurses viewed XR as useful for fear reduction/procedural flow but noted training and communication risks.	No comparative efficacy estimate; context-specific transferability.
71	Tinka et al. (2026) [22]	Routine-practice clinical study	Hungary	children aged 4–18 years	Venipuncture/blood draw	Interactive/active VR distraction	VR significantly reduced venipuncture pain in children, but not adolescents; exploratory benefit was greatest in girls.	Limited confounding control and no long-term follow-up.
72	Storey et al. (2025) [27]	Qualitative/implementation study	Australia	pediatric patients/children; burn care	Burn/wound care procedures	Immersive VR distraction	Patients/parents reported reduced burn-dressing pain/anxiety and high acceptability; clinicians identified fit and age-appropriate-content issues.	No comparative efficacy estimate; context-specific transferability.
73	Worth et al. (2025) [28]	Observational pilot feasibility study	United Kingdom	pediatric patients/children	Venipuncture/blood draw	Immersive VR distraction	Most participating children/parents reported positive, comfortable VR experiences and reuse interest; recruitment reached 41% of target.	Small observational sample; confounding and limited generalizability.
74	Aydos et al. (2024) [86]	Feasibility study	Türkiye	pediatric patients/children	Pediatric procedural care	Biofeedback-based VR game	Golden Breath was feasible, acceptable, and safe; pilot testing showed reduced needle-related pain and fear.	Small feasibility sample; clinical efficacy not established.
75	Girin et al. (2024) [25]	Qualitative/implementation study	United States	pediatric patients/children	Pediatric procedural care	Interactive/active VR distraction	Stakeholders reported high enjoyment and feasibility, reduced observed anxiety, and a need for standardized implementation guidance.	No comparative efficacy estimate; context-specific transferability.
76	Jones et al. (2024) [20]	Randomized clinical trial	United States	children aged 6–17 years; burn care, outpatient	Burn/wound care procedures	Interactive/active VR distraction	Younger patients showed greater analgesic benefit; engagement ratings did not differ by age/sex but related to pain outcomes.	Short-term, single-setting evidence; no powered subgroup analysis.
77	Savaş et al. (2024) [87]	Feasibility study	Türkiye	children aged 6 to 12 years; oncology/hematology	Port/implanted vascular access	Biofeedback-based VR game	BioVirtualPed was feasible, acceptable, and safe; preliminary testing showed reduced pain, fear, and anxiety.	Small feasibility sample; clinical efficacy not established.
78	Savaş et al. (2024) [88]	Randomized controlled trial	Türkiye	children aged 6–12 years; oncology/hematology	Port/implanted vascular access	Biofeedback-based VR game	BioVirtualPed significantly reduced postprocedure pain, fear, and anxiety; respiratory rate was lower and satisfaction higher.	Added value over matched fixed VR was not isolated.
79	Jyskä et al. (2023) [89]	Within-subject feasibility study	Finland	children aged 8 to 12 years	Intravenous cannulation	VR-guided breathing/natural environment	Guided VR breathing significantly reduced HRV-defined physiological stress; no adverse effects occurred.	Small feasibility sample; clinical efficacy not established.
80	O’Sullivan et al. (2023) [90]	Prospective interventional pilot cohort	Ireland	children aged 8–15 years; ED	Pediatric procedural care	VR hypnosis/hypnoanalgesia	Most participants reported lower anxiety, whereas median maximum procedural pain slightly increased from baseline in this uncontrolled pilot.	Pilot sample; underpowered for definitive efficacy or subgroup effects.
81	Whu et al. (2023) [21]	Qualitative/implementation study	Taiwan	children aged 6–12 years	Venipuncture/blood draw	VR education and preparation	Children described VR as helpful for preparation, understanding, and coping with needle fear, with usability caveats.	No comparative efficacy estimate; context-specific transferability.
82	Canares et al. (2021) [26]	Secondary analysis of RCT data	United States	pediatric patients/children; ED	Venipuncture/blood draw	Interactive/active VR distraction	VR was associated with significantly longer venipuncture duration than child-life or reference care.	Exploratory analysis underpowered for moderator effects.
83	Easterlin et al. (2020) [91]	Qualitative/implementation study	United States	pediatric patients/children; outpatient, infusion center	Pediatric treatment stress/needle procedures	Immersive VR distraction	Children/parents generally found VR acceptable for infusions but identified nausea/dizziness, disorientation, and lost coping opportunities.	No comparative efficacy estimate; context-specific transferability.
**G. Recent evidence on developmental modifiers and emerging clinical applications**								
84	Kim et al. (2026) [31]	Integrative literature review	Multinational evidence synthesis	hospitalized children; pediatric nursing settings	Hospital-based pediatric interventions	VR and AR intervention mapping	Across 13 studies, brief VR predominated; pain, anxiety, and fear often improved, with interactive formats tending to perform better.	Mixed VR and AR evidence; broad outcomes and intervention types.
85	Medland et al. (2026) [32]	Narrative review of higher-quality RCTs	Multinational evidence synthesis	children undergoing needle procedures	Needle-related procedural pain	VR and Buzzy; attention to age, analgesia, and design	Across 13 higher-quality RCTs, effects were mixed; benefits were more consistent without concurrent analgesia and in younger children.	Restricted eligibility and needle-focused scope; no formal pooled estimate.
86	Alharran et al. (2026) [33]	Systematic review and meta-analysis	Multinational evidence synthesis	children undergoing orthopedic procedures	Orthopedic procedural pain and anxiety	VR distraction versus control	Meta-analysis of four RCTs found significant reductions in orthopedic procedural pain, anxiety, and heart rate.	Only four RCTs; procedures and VR protocols were heterogeneous.
87	Cocchi et al. (2026) [34]	Bayesian meta-reanalysis	Multinational evidence synthesis	pediatric VR trials; study-level data	Procedural pain and anxiety	Re-analysis of meta-analytic evidence by age	Bayesian re-analysis favored VR for pain and anxiety, with benefit attenuating as study mean age increased.	Study-level age analysis cannot define individual developmental cutoffs.
88	Jyskä et al. (2026) [92]	Feasibility study	Finland	children undergoing IV cannulation	Intravenous cannulation	Mindfulness-guided relaxation in virtual nature	Within-cohort HRV stress was significantly lower during VR relaxation, and experienced pain was significantly below anticipated pain.	Small feasibility sample; efficacy and subgroup effects remain uncertain.
89	Trinh et al. (2026) [93]	Randomized controlled trial	Hungary	adolescents undergoing MIRPE	Perioperative anxiety and postoperative pain	Immersive VR perioperative intervention	VR did not significantly reduce perioperative anxiety; pain was significantly lower only at 1 h postoperatively.	Procedure-specific adolescent sample and short-term endpoints.
90	Gürbüz et al. (2026) [94]	Randomized controlled trial	Türkiye	90 children aged 7–10 years	Skin prick testing	Immersive VR versus Buzzy versus control	Post-test pain, fear, and anxiety differed significantly among groups, with lowest scores for VR.	Single-center study with a narrow age range.
91	Güvener and Kara (2026) [95]	Randomized controlled trial	Türkiye	children receiving inhaler treatment	Inhaler treatment	VR glasses versus routine care	After baseline adjustment, VR significantly reduced post-treatment fear/anxiety and improved several vital-sign measures.	Nonpainful treatment context and limited generalizability to invasive procedures.
92	Zengin et al. (2026) [96]	Randomized controlled trial	Türkiye	42 children aged 5–10 years	Cardiac catheterization preparation	VR educational animated film plus standard care	VR preparation significantly reduced postprocedure fear, anxiety, and pain; postprocedural vital signs did not differ.	Small single-center sample and one content format.
93	Gümüş et al. (2026) [97]	Randomized controlled trial	Türkiye	74 children aged 5–12 years	Preoperative preparation and postoperative pain	VR educational video versus routine preparation	Preoperative fear decreased significantly within the VR group, but did not differ significantly between groups; postoperative pain was significantly lower with VR.	Between-group fear effects were mixed and follow-up was short.
94	Xie et al. (2026) [98]	Randomized controlled trial	China	188 children undergoing IV cannulation	Intravenous cannulation	Immersive VR distraction versus routine care	VR significantly reduced fear at two pre-venipuncture time points; pain showed no significant overall group effect, although nurse-rated FLACC scores showed a significant time-by-group interaction, and dynamic HR and SpO2 changes were significantly lower in the VR group.	Single-center design and limited blinding.
95	Efe et al. (2026) [99]	Randomized controlled trial	Türkiye	60 children aged 7–13 years and their mothers	Preoperative education for day surgery	VR-delivered educational animation	VR education significantly improved child fear/anxiety trajectories and selected heart-rate measures; maternal anxiety showed no added VR benefit.	Single-center trial and short-term outcomes.
96	Felnhofer et al. (2026) [100]	Exploratory follow-up analysis of RCT data	Austria	40 patients aged 10–17 years	Ingrown toenail surgery	Goal-directed versus exploratory immersive VR	Goal-directed VR increased spatial presence/involvement; immersion, enjoyment, comfort, and technology-related adverse effects did not differ.	Small secondary analysis; presence was not tested as a mediator of clinical benefit.

Note: The table summarizes the 96 publications included in the clinical narrative synthesis. Country/region denotes the reported study setting for primary publications; when the recruitment setting was not explicit, the country of the principal clinical institution reported in the publication was used as a transparent proxy. Evidence syntheses were coded as multinational because they summarized studies across settings and were excluded from primary-publication country counts. Contextual References [23,29,37,38], used only to frame technical, cognitive, resource, or institutional considerations, are not included in this table. Findings are reported at a high level to avoid over-interpreting heterogeneous reviews, feasibility studies, implementation research, and clinical trials. The final column summarizes the principal design or reporting limitation and does not constitute a formal risk-of-bias assessment. Abbreviations: ED, emergency department; HMD, head-mounted display; IV, intravenous; RCT, randomized controlled trial; VR, virtual reality; XR, extended reality; PICC, peripherally inserted central catheter; PIV, peripheral intravenous catheter. Main findings report the specific outcomes assessed and distinguish statistically significant between-group effects from non-significant between-group findings, within-group changes, and non-comparative feasibility or qualitative observations where applicable.

**Table 2:** Narrative evidence profile of principal clinical domains and proposed moderators.

Domain or Moderator	Direction and Narrative Consistency	Evidence Basis and Representative Publications	Interpretive Constraint
Needle-related procedures	Frequently favorable versus usual care; mixed versus active distraction or matched non-immersive content.	Multiple reviews and comparative trials [1,2,3,18,32,41,50,63].	Outcome measures, devices, concurrent analgesia, and comparators vary; added value over active alternatives is uncertain.
Burn, oncology, and repeated procedures	Promising but less consistent; repeated use may permit within-child learning.	Reviews, small trials, feasibility, and implementation studies [39,40,76,77,87,88].	Samples are often small and setting-specific; longitudinal benefit is rarely tested.
Age and developmental readiness	Younger samples often show greater average benefit; no validated age cutoff exists.	Secondary analyses, routine-practice data, narrative review, and study-level meta-reanalysis [19,20,22,32,34].	Mostly post hoc or study-level analyses; age cannot substitute for individual developmental assessment.
Baseline anxiety or anxiety sensitivity	May modify immersion and response.	Limited secondary analyses and heterogeneous trials [19,93].	Measures and subgroup definitions are inconsistent; predictive thresholds are unavailable.
Comparator and concurrent analgesia	Observed benefit is generally smaller against active distraction and may vary with analgesic co-intervention.	Direct comparisons and evidence syntheses [15,32,43,63,94].	Comparator content and co-interventions are incompletely standardized.
Content, immersion, and interactivity	Likely influence engagement and fit; no format is consistently superior.	Device comparisons, RCTs, presence analyses, and qualitative studies [18,19,50,70,100].	Hardware and content effects are often confounded and poorly reported.
Preference, tolerance, and workflow	Consistently relevant to acceptability and implementation.	Feasibility, qualitative, implementation, and resource-use studies [21,24,25,26,27,28,55,91].	These designs inform deliverability rather than comparative efficacy.
Biofeedback-responsive VR	Feasible and potentially beneficial in selected contexts.	Small feasibility studies and one procedure-specific randomized trial [86,87,88].	Added value over matched fixed VR has not been isolated.
AI-supported adaptation	No direct comparative pediatric evidence identified.	No direct comparative pediatric publication identified; conceptual context is discussed separately in Section 6.	Future research proposition only; no claim of clinical effectiveness or readiness.

Note: This table is an author-generated narrative synthesis of the included publications. Representative citations are illustrative rather than exhaustive; individual publications may contribute to more than one domain or moderator, so row-wise counts are not additive. Exact corpus counts by dominant analytic role are reported immediately after Table 1. The table reports the dominant evidence type and interpretive constraint but does not constitute a formal risk-of-bias assessment or certainty-of-evidence grade. RCT, randomized controlled trial; VR, virtual reality.

### 2.4. Analytical Framework and Interpretive Weighting

The narrative synthesis was structured around four analytical questions. First, how consistently does immersive VR affect pain, fear, anxiety, distress, cooperation, or procedural acceptability across pediatric settings? Second, which developmental and patient-level factors, including attention, emotional regulation, communication, device tolerance, procedural understanding, baseline anxiety, and previous experience, modify response? Third, which intervention features, including preparation, content, immersion, interactivity, biofeedback, and AI-supported adaptation, determine clinical fit? Fourth, which safety, usability, hygiene, workflow, caregiver, resource, and oversight requirements determine whether VR can be implemented responsibly? Together, these questions define the child–procedure–intervention matching framework used throughout the review.

This analytical structure enabled comparison of clinical effects, developmental response, mechanisms, intervention design, and implementation without implying the certainty of a systematic review or meta-analysis. Direct evidence refers to findings reported in eligible publications. Evidence-informed interpretation refers to relationships inferred by the authors across heterogeneous publications. Conceptual proposal refers to the author-generated matching pathway and future AI-supported decision-support functions. These categories are identified explicitly in the framework, AI, discussion, and conclusion sections. No pooled effect size, formal risk-of-bias grade, or meta-analytic comparison was calculated. Terms such as recurrent or relatively consistent therefore describe the direction of findings across multiple publications; they do not mean high certainty of evidence.

The absence of formal risk-of-bias and certainty assessment materially limits inference. Similar findings across small, unblinded, single-center, feasibility, or exploratory studies may reflect shared design limitations rather than a stable treatment effect. The present review therefore cannot determine whether the magnitude of reported benefit is unbiased, rank procedures by certainty, or support causal practice recommendations. Design-aware interpretation and the limitation column in Table 1 help readers distinguish what each publication can inform, but they are not substitutes for validated appraisal tools. Accordingly, stronger language is reserved for recurrent patterns supported by evidence syntheses and comparative trials, while feasibility, qualitative, biofeedback, and AI-related conclusions are described as preliminary, contextual, or hypothesis-generating.

## 3. Clinical Evidence Across Pediatric Procedural Contexts

### 3.1. Evidence Syntheses and the Overall Direction of Findings

Across the included evidence syntheses, immersive VR is associated with a broadly favorable direction of effect for some pediatric procedural outcomes, but the magnitude, comparative advantage, and certainty of benefit remain unstable across settings. Systematic, scoping, umbrella, integrative, narrative, and re-analytic reviews repeatedly report variation in procedures, age ranges, intervention formats, comparators, outcome measures, and methodological quality [4,5,30,31,32,33,34]. The nursing-focused integrative review further indicates that duration, interactivity, and alignment with the immediate clinical goal are part of intervention design rather than incidental technical details [31]. These publications support clinical promise, not a uniform efficacy claim, and the absence of formal risk-of-bias assessment in the present review prevents certainty-based conclusions.

Procedure-focused syntheses help explain why findings diverge. Reviews of needle-related procedures generally favor VR as a non-pharmacological adjunct but identify inconsistent measurement and weak developmental subgroup analysis [1,2,3,18,41,42,43,44]. A 2026 narrative review reported that concurrent analgesia, age, and study design explained important differences between trials, with more recurrent benefit in younger children [32]. An orthopedic meta-analysis reported modest pooled benefits but included few heterogeneous trials [33], while a Bayesian meta-reanalysis found attenuation of average pain and anxiety effects as study mean age increased [34]. These are literature-supported observations. Converting them into prospective selection, nursing monitoring, stopping rules, and bounded adaptation is the authors’ conceptual contribution and requires prospective validation.

### 3.2. Needle-Related Procedures, Venipuncture, and Intravenous Access

Needle-related care is the largest and most developed area of pediatric procedural VR research. Venipuncture, blood collection, intravenous access, and cannulation are common and usually brief procedures, but they can create considerable anxiety in children. For this reason, they provide suitable clinical conditions for interventions that redirect attention and reduce visual contact with the procedure. Controlled trials, quasi-experimental studies, and smaller clinical investigations have frequently associated VR with lower pain, fear, anxiety, or distress, or with improved procedural comfort, particularly when routine care was used as the comparator [7,49,50,57,58,65,68,70,72,98]. These findings do not mean that VR should be mandatory during needle procedures. However, they support its use as a legitimate option within multimodal pediatric care rather than as an experimental or purely technological novelty.

However, the favorable direction of many studies should not be interpreted as evidence of universal superiority. Several investigations reported mixed effects, no clear advantage over standard care, or outcomes similar to those achieved with simpler forms of distraction [8,15,43,53,64]. Two factors are particularly important in explaining this variation. The first is the comparator. An effect observed against routine care may disappear when VR is compared with an engaging tablet game, a Buzzy device, or the same content presented in a non-immersive format. The second is the intervention itself. Studies described under the general term of VR may differ substantially in interactivity, audiovisual intensity, content, duration, hardware, and level of immersion. A head-mounted immersive system, an interactive game, a semi-immersive dome-screen environment, and a hypnosis program are clinically and psychologically different interventions. The two dome-screen studies [35,36] were therefore interpreted as semi-immersive evidence and were not assumed to be equivalent to head-mounted immersive VR. Treating these modalities as interchangeable weakens interpretation.

The timing and therapeutic purpose of VR create additional differences between interventions. Some systems are used during the painful phase to occupy attention and block visual cues, whereas other systems are introduced before the procedure to explain or rehearse venipuncture or intravenous placement [54,62,92]. Other studies have examined interactive play during IV procedures or non-head-mounted environments designed to be more appropriate for younger children [35,36,61]. Hypnosis-based VR represents another approach because it combines immersion with guided suggestion instead of relying only on distraction [59]. These distinctions should be maintained in future reviews. Combining all needle-related applications into one category can hide why an intervention was effective, which child benefited, and which component was responsible for the observed effect.

### 3.3. Vaccination, Injection, and Routine Procedural Care

Vaccination extends the application of pediatric VR beyond hospital procedures into primary care and routine preventive services. This setting is clinically important because immunization occurs repeatedly throughout childhood, and distress during early encounters may influence later fear, cooperation, and willingness to receive care. Randomized and pilot studies generally indicate that VR can reduce vaccination-related pain, fear, or distress, although effect sizes and definitions of outcomes are not consistent across studies [6,47,48,51]. A pilot study involving needle-phobic children, including children with developmental disabilities, also suggests that tolerability and developmental suitability may be as important as the average treatment effect [55]. These findings are encouraging, but most of the evidence is limited to the immediate procedure. Current studies do not show whether VR changes future vaccine acceptance, anticipatory anxiety at later visits, or long-term avoidance of healthcare.

Studies of injections, catheterization, skin testing, inhaler treatment, and other routine procedures demonstrate why VR must be compared with realistic alternatives. Multisensory stimulation, bubble play, distraction cards, Buzzy devices, and kaleidoscopes can themselves reduce procedural distress [13,14,45,69,81]. Direct comparison during skin prick testing favored VR over Buzzy and routine care, whereas the broader narrative evidence shows that relative benefit changes with concurrent analgesia, age, and study design [32,94]. VR has also reduced fear and anxiety during inhaler treatment, showing that intervention value may extend beyond painful procedures when the clinical goal is regulation of distress [95]. The practical question is whether VR provides enough additional benefit to justify preparation, cleaning, supervision, and staff time. Its added value is most defensible when immersion, preparation, interaction, or regulation addresses a need that a simpler option does not. Clinical selection should therefore be based on added value and patient fit, not technological complexity alone.

### 3.4. Oncology-Related Vascular Access and Repeated Procedures

Pediatric oncology presents a different procedural pattern from most acute-care settings. Children may undergo venous access, intravenous cannulation, implanted port access, and related procedures repeatedly over long treatment periods. In this situation, pain and anxiety can accumulate, and memories of earlier procedures may influence anticipatory fear before the next encounter. A systematic review of implanted vascular access concluded that immersive VR may reduce physical and psychological distress, but it also reported substantial heterogeneity and limited age-specific analysis [40]. Individual studies have similarly reported beneficial effects during catheter or venous procedures, oncology outpatient care, intravenous cannulation, and subcutaneous port access [9,10,73,74,75,76]. These findings indicate promise, but the repeated and longitudinal nature of oncology care requires a different level of evaluation than one-time procedural use.

The repetitive nature of oncology care also provides a strong reason to move beyond one-time passive distraction. Biofeedback-based VR has been evaluated in a feasibility study and in a randomized trial during port catheter needle insertion [87,88]. The evidence is still preliminary, but the design introduces a different model of procedural support. Instead of presenting the same content at every visit, a future system could use the child’s previous tolerance, breathing pattern, engagement, and reported anxiety to guide the next session. Repeated vascular access is particularly suitable for this approach because every encounter generates within-patient information. However, longitudinal personalization cannot be justified only by technical possibility. It must demonstrate meaningful clinical benefit without adding unacceptable burden, reducing acceptability, or making repeated care more complicated over time.

### 3.5. Burn and Wound Care and Other Acute Procedures

Burn and wound care evaluate VR under more demanding conditions than brief venipuncture because dressing changes and hydrotherapy are often longer, more painful, and more difficult to cover through sustained distraction. An updated systematic review and meta-analysis supports a beneficial role for VR in pediatric burn care, while also emphasizing variation between interventions and reported outcomes [39]. Randomized and clinical studies have included adolescents receiving burn care, school-aged children with burn wounds, and young children undergoing hydrotherapy [77,78,79]. Age and engagement may influence response, whereas the available evidence has not shown consistent sex-related differences [20]. Implementation studies further show that acceptability must be considered from the perspectives of patients, caregivers, and clinicians [27]. Evidence from other acute, orthopedic, and perioperative settings is now broader. Interactive VR has been associated with less discomfort during dental injections and laceration repair [11,12], while orthopedic findings remain mixed at trial level but show modest pooled benefit across a small evidence base [16,17,33]. Perioperative trials demonstrate that VR may support procedural preparation, early postoperative pain reduction, or child anxiety, but effects depend on age, baseline anxiety, content, timing, and the selected outcome [93,96,97,99]. Goal-directed content also increases presence more than unguided exploration, directly linking design to engagement [100]. Trials involving ingrown toenail surgery, cast removal, and general procedural care report findings ranging from reduced anxiety or physiological arousal to mixed self-reported pain effects [80,82,84]. Infection-control-compatible goggles have also been examined in pediatric surgical care [83]. Together, these findings show that procedural purpose, duration, access to the clinical field, content design, and infection-control requirements change the practical value of immersion.

### 3.6. Clinical Patterns Emerging from the Evidence

Our cross-publication narrative synthesis yielded three recurrent patterns. First, evidence is concentrated in venipuncture, IV access, injections, and related needle procedures, with more favorable findings against usual care than against active distraction. Second, repeated or demanding care, particularly oncology-related vascular access and burn treatment, appears promising but is supported by smaller and more heterogeneous evidence bases. Third, neutral findings in orthopedic and outpatient settings show that immersion alone does not guarantee added benefit. Across publications, age, anxiety sensitivity, immersion, engagement, device tolerance, prior experience, comparator, and concurrent analgesia emerged as plausible moderators [17,19,20,22,28,32,34], but most were examined post hoc or inconsistently. Table 2 summarizes these patterns without assigning formal certainty grades. The synthesis therefore supports a shift from asking whether VR works on average to testing which VR format, if any, adds value for a particular child, procedure, and realistic comparator.

## 4. Cognitive, Sensory, and Affective Mechanisms of Immersive Virtual Reality

### 4.1. Attentional Competition and Cognitive Engagement

The most common explanation for VR-related analgesia and anxiety reduction is based on the limited capacity of human attention. During a painful procedure, nociceptive input competes with visual threat, anticipatory thoughts, environmental sounds, and close observation of the actions of clinical staff. VR introduces an additional stream of visual, auditory, and interactive information. When this stream is sufficiently engaging, less attentional capacity may remain available for the needle, wound, medical instrument, or expected discomfort. This explanation is consistent with reviews reporting reductions in pediatric pain, fear, anxiety, or distress and also helps explain why the magnitude of the effect differs between studies [1,2,30,44]. However, VR does not eliminate nociceptive input. Its more realistic contribution is to reduce the extent to which that input dominates conscious attention and to change how the child interprets the experience during the procedure. In this context, attention is redistributed rather than removed, while the threatening stimulus remains physically present throughout care.

This attentional explanation also shows why placing a headset on a child is not sufficient by itself. Content that is repetitive, confusing, poorly matched, or excessively passive may fail to maintain attention at the moment when procedural threat becomes strongest. An experience involving visual search, movement, choice, or goal-directed interaction may compete more effectively with pain-related processing. However, excessive cognitive demand can create different difficulties and increase frustration or fatigue. Broader cognitive research demonstrates that task demand, fatigue, perceived effort, attention, and sensory feedback can influence behavioral and physiological responses during challenging activities [38]. This evidence does not directly establish a mechanism for pediatric procedural VR, but it supports a clinically important principle: attention must be captured without exceeding the child’s capacity. Therefore, the relevant issue is not simply whether VR is present. The experience must remain understandable and engaging without producing frustration, fatigue, or sensory overload.

### 4.2. Presence, Immersion, and Perceptual Distance from the Procedure

Attentional distraction alone cannot fully distinguish immersive VR from tablets, videos, kaleidoscopes, or other visual distraction methods. A more specific mechanism is presence, the subjective feeling of being located inside the virtual environment rather than observing it from outside. Presence may create perceptual and emotional distance from the treatment setting by limiting visual access to medical equipment and replacing procedure-related cues with a coherent alternative environment. Greater immersion is not automatically better; its value depends on whether the child experiences the environment as engaging, understandable, safe, and personally relevant. Secondary analysis suggests that high immersion may be especially beneficial for patients with greater anxiety sensitivity, while age and sex were not significant moderators in that dataset. In pediatric burn care, younger patients appeared to obtain greater benefit and engagement was associated with pain-related outcomes [19,20]. A 2026 follow-up analysis further showed that goal-directed VR produced stronger spatial presence and involvement than unguided exploration without reducing comfort [100]. Immersion is therefore not a hardware property alone. It is produced through the interaction between device, content, user, task, and clinical situation.

The format of the device can also change the balance between immersion and clinical practicality. Head-mounted displays can restrict visual contact with the procedure and create a strong sense of environmental substitution. However, they may be less appropriate for very young children or for procedures in which continued visual contact with parents and clinical staff remains important [24,28,36]. Non-head-mounted and dome-screen systems provide a different form of immersion because they preserve greater awareness of the clinical environment while still offering visual engagement. Trials using domed-ceiling or dome-screen environments in young children indicate that meaningful procedural distraction can occur without a conventional headset [35,36]. Evidence comparing different immersive device types also suggests that hardware configuration may influence clinical effects, although current comparisons remain heterogeneous and do not establish one universally superior format [18]. Therefore, the underlying mechanism is better described as effective perceptual engagement rather than simple exposure to a headset.

### 4.3. Anticipatory Anxiety, Procedural Preparation, and Perceived Control

Procedural distress frequently begins before the painful stimulus occurs. Children may anticipate pain, imagine threatening outcomes, interpret unfamiliar equipment as dangerous, or become distressed because they do not know what will happen next. Therefore, VR delivered before a procedure may work through a different pathway from distraction used during needle insertion or wound care. Preparation-oriented VR can present procedural information in a controlled and visually accessible form and allows the child to encounter elements of the clinical sequence before the real event. Randomized studies of VR-based venipuncture education have reported reductions in procedural pain or anxiety. Qualitative studies also show that children may consider interactive VR helpful around intravenous cannulation, although usability and personal preference remain important [21,54,62]. In this context, the clinical purpose is not simply to hide the procedure, but to reduce uncertainty before the procedure starts.

The likely mechanism in preparation-oriented interventions is not limited to distraction. Reduced uncertainty and increased perceived control may be equally important. Familiarity with the sequence of events can make procedural sensations more predictable and reduce the difference between what the child expects and what actually happens. Interactive preparation may also allow the child to rehearse coping behavior or make limited choices about content, timing, or interaction. These forms of control are clinically meaningful because pediatric distress is often intensified by helplessness and the inability to anticipate the actions of staff. Therefore, preparation and distraction should remain conceptually separate. A child who needs information and predictability may benefit more from pre-procedural VR. In contrast, a child who understands the procedure but becomes distressed by the immediate visual or sensory stimulus may benefit more from immersive distraction during the event. Combining both approaches may be useful, but studies should clearly describe the intended therapeutic function instead of labeling every application as VR distraction.

### 4.4. Emotional Regulation, Guided Breathing, Hypnosis, and Biofeedback

Not all pediatric VR interventions are designed primarily as distraction. Some applications use the virtual environment to help the child regulate physiological and emotional arousal. Guided breathing, for example, can be paced through visual or auditory cues within a calming environment and gives the child a concrete task when procedural stress begins to increase. Small feasibility studies using natural virtual environments and breathing guidance have reported greater parasympathetic activity or lower physiological stress during venipuncture and IV cannulation [85,89]. The importance of these studies is that they show how VR can support an active coping behavior rather than functioning only as an audiovisual barrier. However, their limitations are also clear. The samples are small, and a change in physiological arousal should not automatically be interpreted as a clinically meaningful reduction in the child’s subjective pain or fear.

Biofeedback makes the intervention responsive by connecting the virtual environment to a measurable behavior or physiological signal. Pediatric studies have examined breathing-oriented and biofeedback-based games, including applications during port catheter needle insertion in oncology care [86,87,88]. In these systems, the child’s breathing or another physiological signal changes what is seen or experienced in VR. This feedback may help the child recognize increasing arousal and practice a regulatory response, while the resulting change in the virtual environment reinforces successful control. This represents an important shift from passive delivery because part of the experience is directly shaped by the child’s behavior. However, the current evidence remains early and procedure-specific. Biofeedback should therefore be considered evidence that responsive pediatric VR is feasible, not evidence that one particular adaptive method has already achieved clinical validation.

Hypnosis and hypnoanalgesia delivered through VR represent a separate approach to emotional and physiological regulation. In this model, immersion is combined with suggestion, imagery, relaxation, and focused attention rather than ordinary game-based interaction. A non-inferiority trial found that VR hypnosis provided procedural support comparable with conventional medical hypnosis for needle-related pain and fear. A pilot study in an emergency department also reported promising changes in anxiety and pain with VR-delivered hypnotherapy [59,90]. These interventions should not be conceptually combined with game-based distraction because their intended psychological pathway is different. Their effectiveness may depend more strongly on language comprehension, suggestibility, willingness to follow guidance, and the structure and quality of the therapeutic script.

### 4.5. A Multicomponent Rather than Single-Mechanism Intervention

No single mechanism can adequately explain the complete pediatric procedural VR literature. Depending on the intervention, VR may redirect attention, capture the senses, create perceptual distance, improve preparation and predictability, support emotional regulation, pace breathing, strengthen a sense of mastery, or change expectations. The dominant mechanism is likely to vary according to both the child and the procedure. Rapid attentional capture may be central during a short venipuncture. Familiarity and learned coping may become more important across repeated oncology visits. During burn care, the benefit may depend on whether engagement can be sustained without exceeding sensory tolerance. Younger children may respond to simple visual engagement or environmental substitution, whereas older children and adolescents may require greater autonomy, challenge, realism, or personally relevant content. Therefore, VR should be understood as a multicomponent intervention rather than as a single and fixed mechanism.

The current evidence indicates that response may vary according to immersion, anxiety sensitivity, age, engagement, device tolerance, and previous familiarity, while findings related to sex remain inconsistent [19,20,22,28]. This variability helps explain why the same intervention may be helpful for one subgroup but produce little measurable change in another. It also shows the weakness of treating VR as a fixed exposure with the same effect in every pediatric patient. The mechanisms of VR should be interpreted in relation to development and clinical context, not as properties of the headset alone. Therefore, the following section examines how cognition, communication, emotional regulation, procedural understanding, sensory tolerance, autonomy, and content preference change across childhood and how these changes may influence the response to immersive VR.

## 5. Age-Specific and Developmental Responses to Immersive Virtual Reality

### 5.1. Age as a Developmental and Clinical Modifier

Age is reported in almost every pediatric VR study, but it is much less frequently analyzed as a factor that may produce different clinical responses. Preschool children, school-aged children, and adolescents are often combined despite major differences in procedural understanding, attention, emotional expression, communication, sensory tolerance, and digital experience. Reviews have repeatedly identified broad age ranges and weak developmental subgroup analyses as important limitations [5,18,30,32,34]. Chronological age remains clinically informative, but it cannot replace assessment of developmental readiness. Two children of the same age may differ substantially in baseline fear, previous procedural experience, language comprehension, sensory preference, willingness to wear a headset, and ability to interact with virtual content. Age should therefore guide assessment without independently determining intervention selection.

The updated evidence confirms an age effect but rejects a simple cutoff model. Routine-practice data showed clearer venipuncture benefit in children than adolescents, and a pediatric burn trial reported greater analgesic benefit among younger participants [20,22]. The 2026 narrative review likewise found more consistent benefit in younger children [32], while the Bayesian meta-reanalysis demonstrated attenuation of average pain and anxiety benefit as study mean age increased [34]. However, study-level mean-age effects cannot classify an individual child. Other analyses found anxiety sensitivity, immersion, procedure, and comparator to be equally or more informative [17,19]. Age therefore contributes to response prediction, but its clinical meaning depends on developmental readiness, content, baseline distress, procedure, comparator, and patient choice. This is why the framework uses age as one input within matching rather than as a stand-alone eligibility threshold.

### 5.2. Early Childhood and School-Aged Children

Young children present specific practical and developmental challenges. Conventional head-mounted displays may be uncomfortable, difficult to stabilize, or unfamiliar, and complete visual separation from parents and clinical staff may increase distress in some children. Younger patients may also have shorter attention spans, limited understanding of instructions, and greater difficulty communicating dizziness, visual discomfort, fear, or disengagement. In this age group, an effective intervention may depend less on technological complexity and more on immediate visual appeal, predictable content, simple interaction, and continued access to reassurance. Non-head-mounted formats provide one possible alternative. A pilot trial in children aged 2 to 6 years found that a dome-screen environment was feasible during IV placement, although pain differences were not statistically significant in the small sample [36]. A later trial using a domed-ceiling display reported lower observed distress during IV placement in young children [35]. These findings suggest that meaningful engagement can be achieved without complete sensory isolation.

Procedural preparation may be especially important in early childhood because uncertainty itself can produce considerable distress. VR-based venipuncture education has been studied in children aged 4 to 8 years and may help them become familiar with the sequence of events before the painful procedure begins [54]. However, greater immersion should not automatically be interpreted as greater clinical benefit. Among young children undergoing venous cannulation, interactive VR was acceptable but did not reduce pain more than smartphone or tablet distraction [63]. These findings support developmental suitability rather than maximum sensory intensity. For a young child, the best option may be the simplest format that can maintain attention, reduce threatening visual cues, preserve contact with parents and staff, and avoid interference with positioning, observation, or procedural instructions.

School-aged children are represented more frequently than any other developmental group in pediatric procedural VR trials. A large proportion of the evidence on venipuncture, IV placement, vaccination, injection, and port access comes from participants approximately 5 to 12 years of age. Children in this range are often able to follow short explanations, report discomfort, respond to interactive instructions, and remain engaged with goal-directed content. Therefore, both active and passive forms of VR may be feasible. However, this age band is not developmentally uniform. A child entering primary school may differ greatly from a child approaching adolescence in emotional regulation, literacy, digital experience, preferred content, and willingness to accept an adult-selected intervention. Qualitative work around IV cannulation also shows that children can directly identify both the benefits and usability problems of VR [21]. Their perspective should therefore be measured rather than inferred only from parent or clinician observation. This developmental variation must be considered when findings from school-aged samples are generalized to pediatric care.

### 5.3. Adolescents and Developmental Mismatch

Adolescents are commonly included in pediatric samples without being analyzed as a distinct developmental group. This approach incorrectly assumes that they are simply older school-aged children. Adolescents usually have stronger preferences, greater procedural awareness, more experience with digital technology, and a stronger expectation of autonomy. Content designed for younger children may appear simplistic or patronizing and may fail to maintain engagement. Therefore, the limited benefit observed among adolescents in routine venipuncture practice may reflect poor matching between age and content rather than a general failure of immersive VR [22]. In contrast, adolescents undergoing orthopedic pin removal showed a more favorable anxiety response than younger participants, although VR was not superior to tablet distraction overall [17]. The available evidence suggests that adolescent response depends on context, content quality, and choice, not on chronological age alone. Adolescents may also evaluate the credibility, privacy, and relevance of the experience more critically than younger children.

Respect for preference and refusal is an essential part of developmentally appropriate care, particularly during adolescence. One patient may prefer immersive distraction, while another may choose music, a personal device, direct communication with staff, visual observation of the procedure, or no distraction at all. Therefore, offering a genuine choice is part of the intervention and not merely an optional courtesy. When clinical workflow allows, content, interaction, sensory intensity, and timing should be discussed with the patient before use. Baseline anxiety characteristics may also be more informative than age in some situations. In venipuncture research, stronger immersion appeared particularly relevant among individuals with greater anxiety sensitivity [19]. Current evidence is not sufficient to determine the best content for adolescents, but it is sufficient to reject the assumption that one child-oriented program can remain engaging and clinically appropriate across the entire pediatric age range.

### 5.4. Implications for Age-Sensitive Clinical Matching

Developmental readiness must be assessed through more than chronological age. Baseline fear, anxiety sensitivity, previous procedural experience, communication ability, sensory profile, familiarity with immersive technology, and willingness to participate directly influence whether VR is understandable, tolerable, and clinically useful. Comfort, device tolerance, previous experience, and willingness to use VR again are established feasibility determinants [28]. Children with developmental disabilities therefore require individual assessment rather than automatic inclusion or exclusion by age or diagnosis. A pilot immunization study showed that VR can be feasible for needle-phobic children with and without developmental disabilities, while also demonstrating the need for individualized selection [55]. A brief familiarization trial before the procedure can reveal fit more effectively than an age cutoff alone.

Age-sensitive matching does not require rigid age-specific protocols. It uses developmental stage as one input within a broader clinical assessment. Younger children may benefit from short sessions, simple tasks, non-head-mounted formats, and continued parental reassurance. School-aged children may tolerate interactive distraction, procedural rehearsal, guided coping, and limited choice over content. Adolescents require credible content, privacy, autonomy, and genuine control over whether and how VR is used. These are matching principles rather than fixed prescriptions. Direct comparisons of age-tailored formats remain limited, so continuation and adjustment should be determined by the child’s immediate response. Sustained attention, clear communication, and willingness to continue support ongoing use; confusion, discomfort, repeated device removal, withdrawal, or escalating distress require modification or termination.

## 6. Proposed Evidence-Informed Conceptual Framework for Developmentally Informed Personalization and Future AI Support

### 6.1. Evidence Basis and Limits of Fixed VR for Individualized Care

Most pediatric procedural VR interventions are delivered in a fixed format. Children with different developmental profiles, baseline anxiety, procedural histories, sensory tolerances, and coping strategies may receive the same content, intensity, interaction pattern, and duration. Standardization supports reproducibility, but it does not resolve the clinically meaningful variability identified across routine-practice, immersion, anxiety-sensitivity, and feasibility studies [19,22,28]. A fixed program may be appropriate for one child, overwhelming for another, and insufficiently engaging for a third. The limitation is not standardization itself, but the assumption that one standardized experience can fit the pediatric population as a whole.

Personalization does not need to begin with complex AI. It begins with clinical matching: selecting preparation, distraction, guided regulation, interactivity, content, device format, and duration according to the child’s developmental readiness, preference, procedure, and willingness to participate. Technology can then support or refine this decision during the procedure. The objective is not maximum immersion, but sufficient engagement to reduce distress without overwhelming the child or obstructing care. Preference-based selection is therefore a clinically valid personalization strategy and an essential comparator for future adaptive systems.

### 6.2. Operational Definition, Candidate Inputs, and Permitted Outputs

For this conceptual framework, AI-supported personalization means future clinician-facing decision support in which a trained machine-learning model integrates multiple preprocedural and/or intraprocedural inputs and produces one bounded recommendation from a predefined option set. This definition excludes fixed content, preference-based selection, deterministic threshold rules, and single-signal biofeedback. A rule-based system may be adaptive without being AI-based, and a biofeedback loop may be responsive without performing prediction or classification. The distinction is operational rather than promotional: automation should not be labeled AI unless a learned model performs a defined inferential task.

The proposed decision process operates across three time points. Before the procedure, developmental and communication profile, baseline anxiety, sensory sensitivity, previous procedural and VR experience, content preference, procedure characteristics, and the child’s willingness inform initial selection. During the procedure, child report remains the primary subjective source, while behavioral, physiological, and interaction signals provide complementary prompts for reassessment. After the procedure, pain, fear, anxiety, or distress reports must be recorded separately from cooperation, procedural success, tolerance, adverse effects, and willingness to use VR again. In repeated-procedure pathways, these encounter-specific observations could inform the next clinician-led selection. Any data collection must be proportionate, secure, and limited to variables with a defined clinical purpose.

Candidate real-time inputs include child-reported discomfort, breathing, heart rate or heart-rate variability, head and controller movement, gaze, interaction frequency, task performance, verbal response, and attempts to remove the device. These inputs are not equivalent. Validated patient-reported measures capture the child’s subjective experience; behavioral indicators provide observable but non-specific context; physiological measures indicate autonomic arousal; and clinical interpretation integrates the signals with the procedure and direct communication. Increased heart rate may reflect pain, anxiety, excitement, or movement, while reduced movement may indicate relaxation, freezing, withdrawal, confusion, or loss of engagement. No system should be described as detecting pain or anxiety solely from heart rate, movement, gaze, or interaction. Pediatric guided-breathing and physiological-monitoring studies establish technical feasibility [85,89], not diagnostic validity.

Permitted outputs must be defined before evaluation. A future system could recommend an initial content category, lower sensory intensity, simpler interaction, a change in pace or difficulty, breathing guidance, a content switch, or immediate clinical reassessment. It should not diagnose pain or anxiety, independently continue VR despite a stop signal, modify multiple parameters without explanation, or control analgesia, restraint, or the clinical procedure. Every recommendation must be limited, interpretable, reversible, logged, and subject to nurse or clinician approval. If the child reports discomfort, asks to stop, loses safe communication, or shows signs that cannot be interpreted confidently, the correct output is clinical reassessment or termination rather than automated escalation.

### 6.3. Biofeedback as a Pre-AI Model of Responsiveness

Biofeedback provides the clearest current pediatric example of responsive VR. A measurable signal, such as breathing, produces an observable change in the virtual environment and allows the child to practice regulation rather than consume preset content passively. Early feasibility studies and one randomized procedure-specific study indicate that this approach is workable and may reduce selected outcomes [86,87,88]. The evidence remains preliminary, based on limited samples and contexts, and does not establish superiority over matched fixed VR. Biofeedback therefore supports the feasibility of responsiveness, not a general claim of clinical effectiveness and not the validity of AI-based adaptation.

Biofeedback and AI-supported adaptation must remain conceptually separate. Biofeedback uses an explicit signal-response relation that the child and clinician can observe. The future AI concept proposed here would integrate multiple uncertain inputs and estimate whether one predefined recommendation warrants consideration. That additional inferential step creates new requirements for training data, calibration, external validation, subgroup fairness, explanation, privacy, and monitoring. It cannot be justified by biofeedback feasibility alone.

### 6.4. Future AI-Supported Decision Support and Governance

If AI-supported personalization is evaluated, it should remain clinician-facing decision support rather than autonomous control. A model might recommend initial content, flag a pattern consistent with disengagement, or prompt reassessment when several signals change together. Nursing and clinical staff must retain authority to approve, reject, modify, pause, or terminate every recommendation. Governance must specify the clinical purpose, authorized inputs and outputs, accountable professional, override process, audit trail, data minimization, access controls, retention period, model version, performance monitoring, and procedure for managing errors or drift. Assent, parent or guardian permission, and age-appropriate explanation are necessary when identifiable or linkable patient signals are collected. Broader clinical decision-support literature supports these governance principles [101] but does not establish pediatric procedural efficacy.

Evaluation should proceed in stages. Technical studies should first establish sensor reliability and whether proposed inputs have stable meaning in the intended procedure. Feasibility studies should then test usability, workload, communication, stopping rules, and failure handling. Only after those stages should prospective trials compare AI-supported recommendations with fixed VR, preference-based selection, active non-immersive distraction, and usual care while controlling for content and staff attention. Protocols should prespecify inputs, outputs, update frequency, override rules, missing-signal handling, and subgroup analyses. Outcomes should include distinct measures of pain, fear, anxiety, distress, cooperation, procedural success, adverse effects, preference, staff burden, and technical failure.

The reviewed literature supports the clinical rationale for personalization, but it does not demonstrate the effectiveness of any AI technique. Heterogeneity in outcomes, developmental response, immersion, and tolerance shows why one fixed protocol may not fit every child, while biofeedback studies show that limited responsiveness is feasible [86,87,88,89]. The proposed AI role is therefore a hypothesis for evaluation: combine defined child and procedure characteristics with bounded inputs to generate one interpretable recommendation for clinician review. No randomized trial has shown that AI-adaptive VR is superior to fixed VR or preference-based selection. Accordingly, AI-supported adaptation is presented only as a future research and translational opportunity.

**Figure 2:**
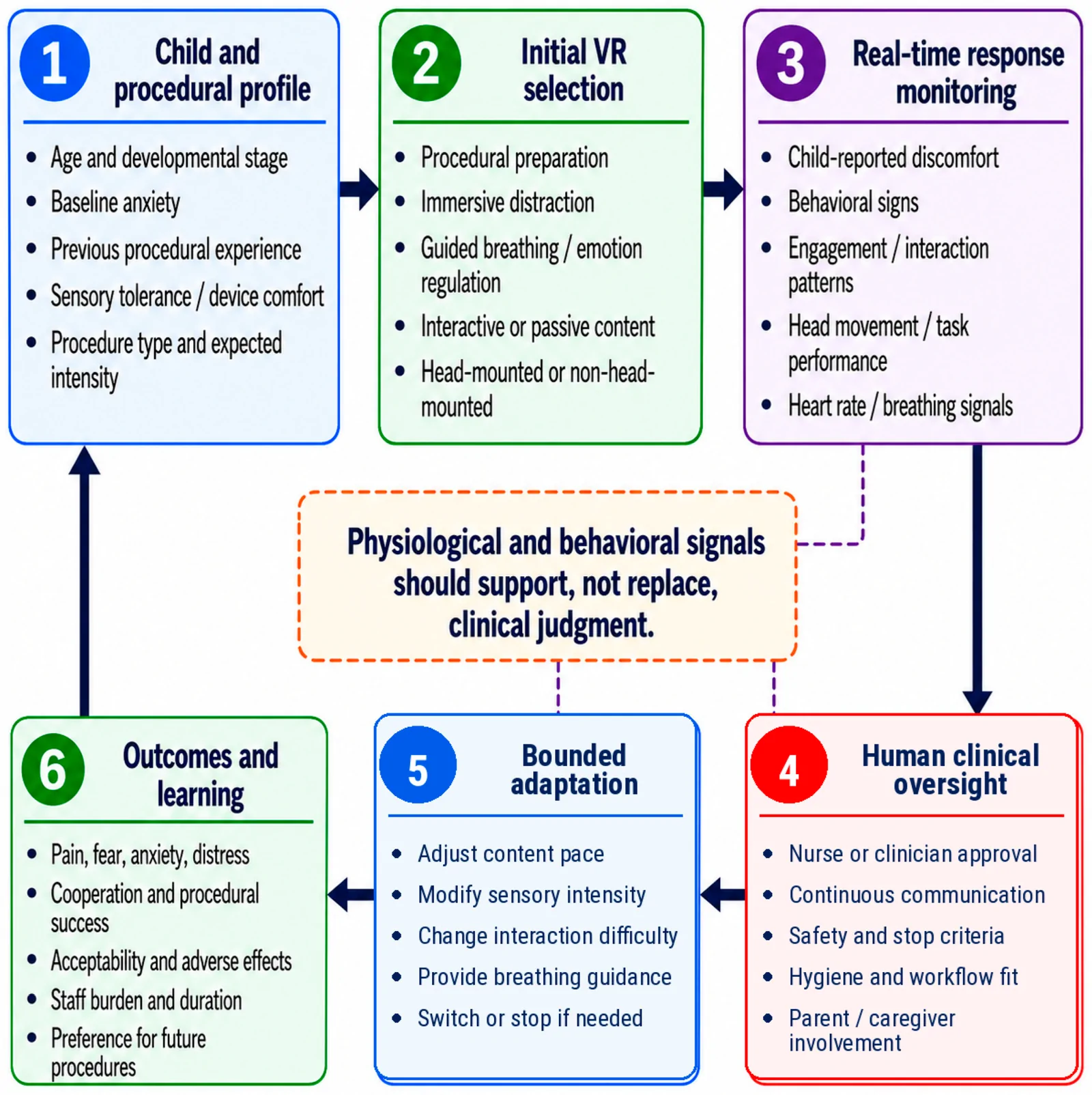
Proposed evidence-informed conceptual framework for developmentally informed clinical matching and clinician-supervised personalization of pediatric procedural virtual reality. The framework is author-generated and has not been prospectively validated. Physiological and behavioral signals prompt clinical review and do not independently identify pain or anxiety; bounded adaptation remains subject to human approval.

Figure 2 presents the author-generated, evidence-informed conceptual framework developed from the narrative synthesis. It is not a validated clinical algorithm. The child and procedural profile informs initial clinician-led VR selection; real-time responses prompt clinical review; approved recommendations may permit bounded adjustment; and staff retain authority over safety, continuation, modification, and termination. Outcomes from each encounter may inform subsequent selection in repeated-procedure pathways. Each link is a testable proposition rather than an established causal or predictive relation.

## 7. Clinical Implementation, Safety, and Workflow Integration

### 7.1. Nursing-Led Workflow and Clinical Accountability

Evidence of efficacy does not guarantee routine adoption. VR adds assessment, content selection, fitting, timing, troubleshooting, cleaning, supervision, and documentation to time-sensitive procedural care. Nursing implementation studies show that successful use depends on preserving communication, assigning responsibilities, maintaining device availability, and embedding VR within the care pathway [24,25]. Table 3 translates the proposed conceptual framework into a six-stage nursing pathway: preprocedural assessment, purpose-based selection, safety preparation, monitoring of distinct outcomes, modification or discontinuation, and documentation for repeated encounters. The pathway is proposed for evaluation and local adaptation; it is not a validated protocol.

Before adoption, each service should designate the nurse or clinician responsible for eligibility assessment, preference elicitation, equipment preparation and cleaning, monitoring, approval of any adjustment, adverse-event documentation, and termination. Staff must retain direct communication with the child and unobstructed access to positioning, lines, airway, and the clinical field. VR should be paused or stopped when it blocks instructions, obscures distress, prevents reassurance, interferes with the procedure, or is rejected by the child. Training should cover developmental matching, outcome distinctions, content selection, communication, cybersickness, stopping criteria, infection prevention, privacy, and technical failure. These responsibilities make nursing assessment and accountability central to the intervention rather than ancillary to the device.

### 7.2. Safety, Tolerability, and Infection Control

Short-term tolerability appears acceptable in many reports, but adverse-event ascertainment is inconsistent and the evidence does not establish safety across all ages, devices, or procedures. Relevant events include cybersickness, nausea, dizziness, headache, eye strain or visual discomfort, disorientation, loss of balance, poor device fit, heat or pressure at facial contact points, sensory overload, panic, and distress caused by reduced awareness of staff or caregivers. Pre-use assessment should include willingness, previous motion sickness or adverse VR experience, sensory and communication needs, device fit, positioning, and the ability to use a clear verbal or non-verbal stop signal. During use, the nurse should maintain communication and observe for pallor, sweating, repeated device adjustment, withdrawal, reduced responsiveness, unsafe movement, escalating distress, or attempts to remove the device. Symptoms, a stop request, impaired communication, or interference with care require prompt reassessment and, when necessary, immediate removal [28,83,89].

Infection prevention is an independent operational requirement because facial interfaces, straps, controllers, headphones, and headset surfaces may contact skin, hair, respiratory secretions, or hands and may be shared between patients. Each service should use a written protocol approved by its infection-prevention team and compatible with the manufacturer’s instructions. The protocol should specify which components are single-patient, disposable, removable, or reusable; the cleaning agent and required contact time; management of porous straps and difficult-to-clean interfaces; drying and storage; inspection for material damage; hand hygiene; responsibility for documentation; and criteria for removing equipment from service. Removable or disposable facial interfaces may simplify turnover but do not replace decontamination of other touched surfaces. Infection-control-approved goggles in pediatric surgical care show that hardware selection should begin with clinical hygiene requirements rather than assumptions that consumer devices are suitable [83]. Services caring for immunocompromised children should obtain explicit local infection-prevention approval before shared-device use.

### 7.3. Acceptability, Accessibility, and Developmental Inclusion

Acceptability is created by the interaction among the child, caregiver, clinical team, device, content, and procedure. Children may value distraction, reduced exposure to threatening cues, novelty, and participation, while caregivers may value calmer cooperation. Others may dislike the headset, lose access to direct reassurance, or disengage when content does not match their interests or developmental capacity. Qualitative studies around IV cannulation and infusion care show that children and families can identify both benefits and practical barriers, and their views must inform selection and evaluation [21,91]. Children should be free to decline or discontinue VR at any point without compromising access to appropriate procedural care. Acceptability must be reassessed at every encounter rather than assumed from a previous successful session.

Developmental inclusion requires more than the definition of a lower age limit. Children with developmental disabilities, communication differences, sensory hypersensitivity, motor limitations, or high needle fear may benefit from VR, but they may also face barriers that are not reflected by chronological age. A pilot immunization study found VR feasible and acceptable for many needle-phobic children with and without developmental disabilities. However, the study also showed that controlled evaluation and individualized assessment remain necessary [55]. Alternative formats may improve access when head-mounted systems are poorly tolerated. Dome-screen and projected environments can provide visual engagement while preserving awareness of parents and staff, although these systems also require space, equipment, and compatibility with the procedure. Therefore, accessibility should be evaluated in relation to communication, sensory profile, positioning, vision, hearing, physical interaction, and the demands of the specific clinical procedure. An intervention that excludes the children who experience the greatest procedural distress has limited clinical value, even when average outcomes are favorable in groups that are easier to treat.

### 7.4. Resource Use, Scalability, and Implementation Standards

The cost of VR extends beyond the headset to software licensing, replacement parts, disposable interfaces, cleaning, storage, charging, staff training, technical support, content updates, and preparation time. A pediatric venipuncture resource-use analysis found that VR can alter staff and procedural resource requirements and may prolong the procedure [26]. References [29,37] concern other technology-intensive clinical services and are cited solely as cross-domain context for generic cost categories and institutional readiness; they do not demonstrate the cost-effectiveness, feasibility, or sustainability of pediatric VR. Pediatric VR services must evaluate these questions directly in their own settings. Economic assessment should therefore include staff time, number of attempts, procedure duration, caregiver burden, technical failure, equipment life, and the opportunity cost of setup and turnover.

Scalable implementation requires a protocol that standardizes safety-critical steps while preserving individualized clinical matching. The protocol should define eligible procedures, contraindications, device and content options, fitting and cleaning procedures, staff roles, stopping criteria, adverse-event recording, and the preferences of the child and caregiver. A local pilot should measure reach, uptake, fidelity, preparation time, technical failure, workload, acceptability, refusal, and discontinuation before wider adoption. Stakeholder studies show that sustainability depends on integration into routine practice rather than reliance on a small number of enthusiastic users [24,25,27]. The implementation standard is therefore straightforward: VR should be adopted only when the service can deliver it safely, consistently, and without displacing essential nursing or procedural care.

## 8. Discussion, Limitations, and Future Directions

### 8.1. Integrated Interpretation of the Evidence

The direct literature reviewed here suggests that pediatric procedural VR may have conditional rather than uniform clinical value. Variation across publications reflects differences in procedures, comparators, concurrent analgesia, devices, content, interactivity, timing, baseline distress, developmental readiness, sensory tolerance, and patient preference [1,2,3,5,30,31,32,33,34]. Our interpretation is that VR should be treated as a configurable procedural intervention whose value depends on the match among the child, clinical task, therapeutic purpose, and intervention design. This matching interpretation is evidence-informed but remains a conceptual synthesis rather than a validated predictive model.

Neutral or negative findings are equally informative. Failure to outperform a tablet, an active distraction device, or well-delivered routine care does not show that VR lacks clinical value; it shows that added value depends on the comparator and the specific need addressed [16,43,64]. VR is justified when its immersive, interactive, preparatory, or regulatory properties provide an advantage that a simpler alternative cannot provide. This comparative interpretation prevents technological novelty from being mistaken for clinical benefit.

Chronological age alone is insufficient for intervention selection. The updated evidence consistently shows stronger average benefit in younger samples, including the narrative synthesis and Bayesian meta-reanalysis published in 2026 [32,34]. That pattern is clinically important, but it does not justify rigid age thresholds because studies also identify anxiety sensitivity, immersion, procedure, content, and comparator as response modifiers [17,19,20,22]. Age must be interpreted together with communication ability, developmental readiness, procedural history, baseline anxiety, sensory tolerance, technology familiarity, and patient preference. Clinical matching and meaningful patient choice should precede more complex adaptation.

### 8.2. Methodological Limitations of the Current Evidence Base

The current evidence base remains difficult to compare because studies differ in procedure, setting, sample size, age range, hardware, content, interaction, timing, comparator, duration, and outcome measurement. Pain, fear, anxiety, distress, cooperation, arousal, acceptability, and satisfaction are assessed with different instruments and at different time points. Most studies report only immediate outcomes and provide little information on anticipatory distress, memory of the procedure, willingness to return, or response during subsequent procedures. Small samples and single-center designs further limit precision, particularly in burn care, oncology, developmental disability, and biofeedback research. The field now needs better-designed comparisons that answer specific clinical questions rather than additional isolated trials with incompatible methods [4,5,18].

Comparator selection is a major source of uncertainty. A comparison with unstructured routine care estimates a different effect from a comparison with an active distraction method or with identical content delivered on a tablet. Future studies should separate the contributions of content, immersion, interactivity, novelty, and staff attention. Hardware, field of view, audio, interaction method, timing, familiarization, staff involvement, content characteristics, and reasons for discontinuation must be reported consistently. Without this information, apparently conflicting findings may reflect poorly described intervention differences rather than genuine disagreement about clinical effectiveness.

Developmental analysis is another major weakness. Broad pediatric samples are frequently analyzed as one group despite substantial differences in cognition, emotional regulation, communication, sensory processing, and technology use. Post hoc age analyses are usually underpowered and rarely include developmental disability, baseline fear, previous procedural experience, or content preference. Child self-report, parent report, and clinician observation should also be analyzed separately because they capture different components of the procedural experience. The field does not need further general statements that VR works in children. It needs prospectively defined estimates of which children benefit, from which VR format, during which procedure, and compared with which realistic alternative.

### 8.3. Limitations of This Review

This review has important limitations. It was designed as a structured narrative synthesis rather than a systematic review or meta-analysis; therefore, no pooled estimate, formal risk-of-bias assessment, or certainty grading was performed. This omission means that recurrent favorable findings may still be influenced by lack of blinding, small samples, selective reporting, single-center recruitment, inconsistent comparators, or other design limitations. The review cannot determine the certainty or unbiased magnitude of effect, and its conclusions should not be used as graded practice recommendations. The explicit multi-database search, documented screening pathway, structured evidence table, design-aware interpretation, and narrative evidence profile improve transparency but do not substitute for formal appraisal.

The search was restricted to English-language publications from January 2020 to 31 July 2026. The date restriction was intended to emphasize contemporary portable hardware, interactive content, accessibility, and implementation, but 2020 is not a definitive technological boundary. Excluding pre-2020 primary studies may omit foundational trials, longer follow-up, early device comparisons, and evidence needed to interpret historical development. Although eligible recent reviews incorporate parts of the earlier literature, they do not replace direct extraction and critical reading of every earlier primary report. The English-language restriction creates a risk of language and selection bias because eligible studies published in other languages, regions, or locally indexed journals may differ in intervention delivery or findings. Both restrictions may distort the apparent direction of evidence and reduce geographic and cultural transferability. Citation chaining and targeted retrieval broadened coverage, but these methods are less reproducible than database searching and do not eliminate these limitations.

The included publications answered different questions and were interpreted according to design rather than treated as one homogeneous evidence base. Randomized and other comparative studies informed short-term clinical effects; feasibility studies informed deliverability and short-term tolerability; implementation studies informed workflow; qualitative studies informed patient, caregiver, and staff experience; safety and usability reports informed operational precautions; and evidence syntheses informed recurrence and heterogeneity across settings. These design-specific roles do not constitute quality or certainty grades, and evidence of feasibility, acceptability, safety, usability, or implementation was not treated as proof of clinical effectiveness. Broader contextual References [23,29,37,38] supported only bounded technical, cognitive, resource, or institutional statements and were not treated as pediatric VR evidence. No randomized trial has demonstrated superiority of AI-adaptive VR over fixed VR or preference-based selection.

### 8.4. Priorities for Future Research and Clinical Translation

Future trials should use clinically informative comparators. A strong design would compare fixed VR, preference-based selection, adaptive VR, an active non-immersive comparator, and usual care while controlling for content and staff attention. Multicenter recruitment would improve generalizability, and developmental groups should be defined before analysis. Baseline anxiety, previous procedural experience, VR familiarity, sensory sensitivity, content preference, communication ability, and chronological age should be assessed prospectively. Repeated-procedure settings, including oncology and chronic treatment pathways, are particularly valuable because they allow evaluation of learning, habituation, anticipatory distress, and patient-specific response across encounters.

Outcome assessment should extend beyond immediate pain and anxiety. Trials should include child-reported fear or distress, behavioral observation, procedural success, number of attempts, procedure duration, restraint or additional support, adverse effects, patient preference, caregiver experience, and staff workload. Follow-up should determine whether VR changes anticipation of the next procedure and willingness to return. Economic evaluation should include setup, cleaning, licensing, technical support, staff time, and equipment life. Reproducibility requires complete reporting of hardware, software, content, interaction, timing, staff involvement, and stopping criteria.

Adaptive and AI-enabled VR should be developed stepwise. Before any signal is used to change the intervention, its technical reliability and clinical meaning must be established. Heart rate, respiration, gaze, head motion, interaction frequency, and task performance may provide useful context, but none directly measures pain or anxiety. Adaptation rules should be predefined, bounded, interpretable, reversible, and overrideable by clinicians. Models require external validation across ages, devices, procedures, settings, and children with developmental or communication differences. Privacy, data minimization, assent, parental or guardian consent, and transparent explanation of how patient signals influence the system must be built into development from the outset.

Clinical translation should proceed from the simplest effective form of personalization to more complex adaptation. Patient choice is already a valid clinical strategy and the essential benchmark before AI is introduced. Biofeedback provides the intermediate model because it links a defined patient signal to a visible and interpretable system response [86,87,88]. Adaptive tools should enter practice only after demonstrating added clinical value, manageable workload, developmental fairness, safety, and workflow compatibility. The objective is not to build the most technologically advanced VR system, but to build the system that best fits the individual child, the procedure, and the clinical team.

## 9. Conclusions

Immersive VR should not be treated as a uniform intervention in pediatric procedural care. The most recurrent favorable evidence concerns needle-related procedures, especially against usual care, while comparative advantage over active distraction and evidence in other settings remain variable. Pain, fear, anxiety, distress, cooperation, physiological responses, acceptability, and procedural success are distinct outcomes and should be measured and interpreted separately. This review proposes an author-generated, evidence-informed conceptual framework linking nursing assessment, purpose-based selection, monitoring, stopping criteria, and clinically approved adjustment. The framework is hypothesis-generating and requires prospective validation before clinical adoption. Biofeedback provides preliminary evidence that responsive pediatric VR is feasible in selected contexts. AI-supported adaptation has not demonstrated clinical effectiveness and should remain a future research and translational opportunity governed by defined inputs, bounded recommendations, privacy safeguards, external validation, and continuous human authority. The immediate clinical priority is not maximum immersion or automation, but the safest acceptable intervention that adds value for the individual child and procedure.

## Figures and Tables

**Table 3:** Operational nursing pathway derived from the proposed conceptual framework.

Stage	Nursing Assessment or Action	Decision or Output	Safety and Evidence Boundary
1. Preprocedural assessment	Elicit willingness and preference; assess developmental and communication profile, baseline fear or anxiety, previous procedural and VR experience, sensory tolerance, motion-sickness history, device fit, and procedure requirements.	Proceed with VR, offer an alternative, or defer use.	Chronological age alone is insufficient. Refusal or inability to maintain communication overrides technological eligibility.
2. Select purpose and format	Choose preparation, distraction, guided regulation, or no VR; select head-mounted or semi-immersive format, content, interaction, timing, and duration.	Use the simplest acceptable option matched to the child and procedure.	These are clinician-led matching decisions; they are not validated AI outputs.
3. Prepare and establish safety	Confirm equipment function, positioning, access to the clinical field, infection-control readiness, a verbal or non-verbal stop signal, and responsibility for monitoring.	Start only when communication and stopping procedures are clear.	The device must not obstruct essential care, observation, or emergency access.
4. Monitor distinct outcomes	Prioritize child report; observe behavior and communication; use physiological or interaction signals only as complementary context.	Continue, pause for reassessment, or modify one bounded parameter.	Behavioral and physiological changes are non-specific and must not be labeled pain or anxiety without direct assessment.
5. Modify or discontinue	Respond to reported discomfort, cybersickness, confusion, withdrawal, escalating distress, blocked communication, unsafe movement, or procedural interference.	Clinician-approved adjustment, device removal, or termination.	A stop request or safety concern requires immediate action; no automated recommendation can override it.
6. Document and learn	Record pain, fear, anxiety, distress, cooperation, procedural success, adverse effects, acceptability, interruptions, workload, and future preference as separate outcomes.	Inform the next encounter and local quality improvement.	Repeated-use learning remains clinician supervised; secondary use of data requires defined governance and permission.

Note: This pathway is an author-generated, evidence-informed translation of the narrative synthesis. It is intended for prospective evaluation and local protocol development, not as a validated clinical guideline. VR, virtual reality.

## Data Availability

No new primary data were generated. The literature-identification pathway and the extracted characteristics of the included publications are reported within the article.

## Public Involvement Statement

No public involvement occurred in any aspect of this research.

## Guidelines and Standards Statement

No dedicated reporting guideline for structured narrative reviews was identified in the EQUATOR Network. Accordingly, this manuscript was prepared using an explicit, predefined framework for transparent narrative evidence synthesis, including a clearly specified review objective, documented multi-database search strategy, eligibility criteria, study-selection pathway, structured evidence characterization, critical interpretation according to study design, and explicit reporting of limitations and clinical implications.
